# Distinct Roles of Matrigel Enabled the Production of Expandable Hepatoblast and Polarized Hepatocyte Organoids from Human Embryonic Stem Cells under 3-Dimensional Suspension Conditions

**DOI:** 10.34133/bmr.0280

**Published:** 2025-11-07

**Authors:** Haibin Wu, Jue Wang, Shoupei Liu, Yiyu Wang, Jinghe Xie, Xueyan Zhang, Shuai Zhang, Weili Gu, Yongjian Zhou, Yuyou Duan

**Affiliations:** ^1^ Department of Gastroenterology and Hepatology, Guangzhou Digestive Disease Center, The Second Affiliated Hospital, School of Medicine, South China University of Technology, Guangzhou 510006, China.; ^2^ Laboratory of Stem Cells and Translational Medicine, Center for Medical Research on Innovation and Translation, Institute of Clinical Medicine, The Second Affiliated Hospital, School of Medicine, South China University of Technology, Guangzhou 510006, China.; ^3^ Laboratory of Stem Cells and Translational Medicine, Institute for Life Science, School of Medicine, South China University of Technology, Guangzhou 510006, China.; ^4^ School of Biomedical Sciences and Engineering, South China University of Technology, Guangzhou International Campus, Guangzhou 511442, China.; ^5^ Department of Gastroenterology and Hepatology, Guangzhou Digestive Disease Center, Guangzhou First People’s Hospital, Guangzhou 510180, China.; ^6^ Surgical Department of Hepatobiliary Pancreas and Spleen, Affiliated Chinese Medicine Hospital at Tianhe Campus, Guangzhou Medical University, Guangzhou 510645, China.; ^7^ National Engineering Research Center for Tissue Restoration and Reconstruction, South China University of Technology, Guangzhou 510006, China.; ^8^ The Innovation Centre of Ministry of Education for Development and Diseases, The Second Affiliated Hospital, School of Medicine, South China University of Technology, Guangzhou 510006, China.

## Abstract

The liver is essential for a range of metabolic functions, and recent advances in organoid technology have enabled the development of various liver organoids. However, the challenges of replicating the in vivo microenvironment, particularly the extracellular matrix, remain important in liver organoid culture. In this study, we explored the roles of Matrigel, a commonly used extracellular matrix component, in the expansion, polarization, and functional maturation of liver organoids derived from human embryonic stem cells (hESCs) under 3-dimensional suspension conditions. Using multiple assays, we demonstrated that a low concentration of Matrigel supported the efficient expansion of hESC-derived hepatoblast organoids by regulating reactive oxygen species (ROS)–autophagy homeostasis through the inhibition of ROS–AMPK–mTOR-mediated excessive autophagy. Moreover, Matrigel induced the polarization of mature hepatocyte organoids differentiated from hESC-derived expandable hepatoblast organoids via activation of the FAK–ERK–AMPK pathway under 3-dimensional suspension conditions. Our findings underscore the importance of integrin signaling in hepatocyte organoid culture and provide insights for the development of artificial, synthetic, bioactive hydrogels for the large-scale production of hepatocytes for clinical applications.

## Introduction

The liver is a vital organ that performs numerous essential functions, including detoxification, metabolism, and protein synthesis. It plays a central role in maintaining the body’s homeostasis and supporting overall health [1]. Hepatocytes, the primary cell type in the liver, are responsible for executing a wide range of biochemical processes. With the advancement of organoid technology, various liver organoids have been developed from primary human hepatocytes (PHHs) and human pluripotent stem cells. These include hepatocyte organoids [2], cholangiocyte organoids [3], hepatobiliary organoids [4], and multilineage hepatic organoids [5]. The creation of liver organoids has opened new opportunities for studying liver development, disease modeling, drug testing, and regenerative medicine [6].

Hepatocytes exhibit a highly polarized architecture in vivo [7,8], which is essential for the secretion and uptake of bile and other components. Polarized hepatocytes are typically cultured in vitro using specialized methods, such as the collagen sandwich culture model [9] or the transwell culture system [10], both of which are employed under 2-dimensional (2D) culture conditions. However, the microenvironmental signals required for the growth, expansion, and polarization of hepatocyte organoids remain largely unknown. Liver organoid cultures often rely on high concentrations (>50% vol/vol) of Matrigel (MG), which is derived from a murine sarcoma cell line and enriched with biologically active extracellular matrix (ECM) proteins [11]. MG supports the long-term culture and function of liver organoids [12,13]. Due to the animal-derived origin of MG, there is growing interest in developing synthetic bioactive hydrogels as substitutes for MG [14–16]. However, the complexity and variability of the composition of MG make it challenging to replicate its function with artificial hydrogels. Understanding the critical signaling interactions between MG and organoids is essential for the development of synthetic bioactive hydrogels [17].

Recently, we developed a stepwise protocol to generate expandable hepatoblast organoids (HB-orgs) and mature polarized hepatocyte organoids (P-hep-orgs) from human embryonic stem cells (hESCs) under 3-dimensional (3D) suspension culture. In this protocol, hESCs were first differentiated into definitive endoderm (DE) and then into hepatoblast (HB) spheres, which were subsequently reaggregated and expanded in HB-expansion medium with a low concentration of MG. We demonstrated that adding 5% (vol/vol) growth-factor-reduced MG to the suspension culture was essential because it promoted efficient expansion of HB-orgs and supported the establishment of apical–basal polarity and functional maturation in P-hep-orgs, as confirmed by structural and functional analyses as well as single-cell RNA sequencing (RNA-seq), and also enabled the large-scale production of both HB-orgs and P-hep-orgs in spinner flasks [18]. In this study, by comprehensively comparing HB-orgs and P-hep-orgs cultured with or without MG, we analyzed the function of MG-triggered integrin signaling in the expansion of HB-orgs and the polarization and functional maturation of P-hep-orgs. To dissect the roles of MG in these processes, multiple approaches, including RNA-seq analysis and small-molecule inhibitors, were used, and the functions and polarized structures of organoids were further assessed.

## Materials and Methods

### Cell culture

The hESC line H9 was obtained from WiCell Research Institute (Madison, WI, USA) under Materials Transfer Agreements (Nos. 19-W0512, 24-W0162, and 24-W0163). H9 cells were cultured on mouse embryonic fibroblast feeder layers in Dulbecco’s modified Eagle medium/F12 medium (Gibco, C11330500BT), containing 20% knockout serum replacement (KSR; Gibco, 10828028), 1% nonessential amino acids (Gibco, 11140050), 0.1 mM 2-mercaptoethanol (Sigma, M3148), 1% GlutaMAX I (Gibco, 35050061), and 10 ng/ml basic fibroblast growth factor (PeproTech, 100-18B). H9 cells were maintained in a humidified incubator at 37 °C with 5% CO_2_. The culture medium was changed daily, and H9 colonies were dissociated into small clumps by collagenase IV (Gibco, C0130) and transferred onto fresh feeder layers every 5 to 6 d.

HepG2 cells were obtained from the Cell Bank of the Chinese Academy of Sciences (Shanghai, China) and cultured as described in the protocol from the provider. PHHs were isolated from excess liver tissues of surgical liver resections from patients, and the use of PHHs was approved by the Research Ethics Committee of Guangzhou First People’s Hospital (Ethical Approval No.: K-2019-167-02). A modified 2-step collagenase perfusion procedure was used to isolate hepatocytes. PHHs were snap-frozen immediately after the isolation, and the frozen PHHs were thawed and seeded onto 6-well plates coated with type I collagen (Corning, C0130) in hepatocyte culture medium (HCM; Lonza, CC-3199) supplemented with SingleQuots (Lonza, CC-4182) minus epidermal growth factor (EGF), 1% B27 (Gibco, 17504044), 100 nM dexamethasone, 20 ng/ml fibroblast growth factor 4 (FGF4), 20 ng/ml hepatocyte growth factor (HGF), and 40 ng/ml oncostatin M (PeproTech, 300-10) when used.

### Differentiation of HB-orgs from hESCs under 3D suspension culture conditions

To generate H9 aggregates in a 3D suspension culture, H9 colonies were dissociated into single cells using the Gentle Cell Dissociation Reagent (Stem Cell Technologies, 100-0485) and seeded into ultralow-attachment 6-well plates (Corning, 3471) at a density of 2 × 10^5^ cells/ml in mTeSR1 (Stem Cell Technologies, 85850) with 10 μM Y27632 (MedChemExpress, HY-10071) for 1 d in a humidified incubator at 37 °C with 5% CO_2_. The medium was changed daily, and the single cells formed aggregates spontaneously after 3 d. After 3 d, for the generation of DE spheres [19], H9 aggregates were washed and cultured in RPMI 1640 medium (Gibco, C11875500BT) supplemented with 100 ng/ml activin A (PeproTech, 120-14) and 3 μM CHIR99021 (MedChemExpress, HY-10182) for 1 d and then transferred to RPMI 1640 medium supplemented with 100 ng/ml activin A and 0.8% KSR for the second day, and the concentration of KSR was 8% on the third day. DE spheres were then transferred to a hepatoblast differentiation medium consisting of Iscove’s modified Dulbecco’s medium (IMDM; Gibco, C12440500BT), 20% fetal bovine serum (ExCell Bio, FND500), 1% GlutaMAX I, 0.3 mM 1-thioglycerol (Sigma, M6145), 100 nM dexamethasone (Sigma, D4902), 0.5% dimethyl sulfoxide (MP Biomedical, 196055), 0.126 U/ml human insulin (Sigma, 91077C), 20 ng/ml FGF4 (PeproTech, 100-31), 20 ng/ml HGF (PeproTech, 100-39), 10 ng/ml bone morphogenetic protein 2 (BMP2; PeproTech, 120-02), and 10 ng/ml BMP4 (PeproTech, 120-05) and cultured for 6 d, and the medium was replaced daily.

To generate HB-orgs, differentiated HB spheres were dissociated into single cells with TrypLE (Gibco, 12604021), and single cells were seeded into ultralow-attachment 6-well plates at a cell density of 2 × 10^5^ cells/ml and cultured in HB-expansion medium that consisted of IMDM, 10% fetal bovine serum, 1% insulin–transferrin–selenium–ethanolamine (Neobioscience, 00-101-10), 1% nonessential amino acids, 1% GlutaMAX I, 0.3 mM 1-thioglycerol, 10 mM nicotinamide (Sigma, N0636), 5 μM CHIR99021 (MedChemExpress, HY-10182), 10 μM SB431542 (MedChemExpress, HY-10431), 10 μM forskolin (MedChemExpress, HY-15371), 20 ng/ml BMP4, 20 ng/ml FGF4, 20 ng/ml EGF (PeproTech, AF-100-15), and 10 μM Y27632 for 1 d, and cells reaggregated to form spheres within 24 h. Next day, the culture medium was supplemented with 5% (vol/vol) growth-factor-reduced MG (Corning, 354230). The medium was changed daily for 5 to 6 d and the typical morphology of HB-orgs would emerge within 5 d. For long-term passaging of the organoids, HB-orgs were dissociated into single cells with TrypLE and split 1:4 to 1:6 every 5 to 6 d, and 5% (vol/vol) ice-cold growth-factor-reduced MG was added when seeding cells at each passage.

### Differentiation of HB-orgs into P-hep-orgs

HB-orgs were directly cultured in HCM supplemented with SingleQuots minus EGF, 1% B27, 100 nM dexamethasone, 20 ng/ml FGF4, 20 ng/ml HGF, and 40 ng/ml oncostatin M. At the start of culture in HCM, 5% (vol/vol) ice-cold growth-factor-reduced MG was added to the medium. The medium was changed every other day for at least 6 d.

### Analysis of cell viability

Equal numbers of HB-orgs were differentiated into P-hep-orgs and nonpolarized hepatocyte spheres (NP-hep-spheres), respectively. At day 12, cell viability was assessed using the CellTiter-Glo 3D Cell Viability Assay (Promega, G9681) following the manufacturer’s instructions. Experiments were performed in 3 independent replicates.

### Assessment of the cytotoxicity of drugs using P-hep-orgs

For cytotoxicity assessment, around 10 to 20 of P-hep-orgs on day 6 were transferred per well into ultralow-attachment 96-well plates (Corning, 3474). The tested compounds were dissolved in dimethyl sulfoxide depending on their solubility to prepare working solutions with a series of concentrations, including mannitol (0, 0.1, 1, 10, 100, 1,000, 2,000, and 10,000 μM; MedChemExpress, HY-N0378), troglitazone (0, 0.1, 1, 5, 10, 50, 100, 500, and 1,000 μM; MedChemExpress, HY-50935), chlorpromazine (0, 0.01, 0.05, 0.1, 1, 10, 50, 100, and 1,000 μM; Selleck, S5749), diclofenac (0, 0.1, 1, 5, 10, 50, 100, 1,000, and 2,000 μM; MedChemExpress, HY-15036), cyclosporine A (0, 0.1, 1, 5, 10, 50, 100, 1,000, 2,000, 5,000, and 10,000 μM; MedChemExpress, HY-B0579), nefazodone (0, 0.01, 0.1, 1, 5, 10, 50, 100, 500, 1,000, and 2,000 μM; MedChemExpress, HY-B1396), tolcapone (0, 0.1, 1, 5, 10, 50, 100, 1,000, and 2,000 μM; MedChemExpress, HY-17406), and bosentan (0, 0.1, 1, 10, 25, 50, 100, 500, 1,000, and 2,000 μM; MedChemExpress, HY-A0013). The cell viability was measured using the CellTiter-Glo 3D Cell Viability Assay 48 h after compound exposure, according to the manufacturer’s instructions. Normalized dose–response curves and TC_50_ values were plotted using the GraphPad Prism software. Cholestasis was assessed by staining with 2 μM 5(6)-carboxy-2′,7′-dichlorofluorescein diacetate (CDFDA; Sigma, 21884) for 1 h at 37 °C. Organoids were rinsed 3 times with phosphate-buffered saline (PBS), and fluorescence images were acquired by single-photon confocal microscopy.

### Quantitative reverse transcription polymerase chain reaction

Total RNA was extracted using the RNAiso Plus kit (Takara, 9109) according to the manufacturer’s instructions. One microgram of RNA was reverse-transcribed into complementary DNA using the PrimeScript RT Master Mix (Takara, RR036B). Quantitative reverse transcription polymerase chain reaction (qRT-PCR) was performed in triplicate using PowerUp SYBR Green (Thermo, A25742) on the QuantStudio 1 Real-Time PCR system (ABI, Thermo, USA). The cycle threshold (CT) values for each sample were normalized to the expression of the housekeeping gene glyceraldehyde-3-phosphate dehydrogenase. Relative gene expression levels were calculated using the 2^−ΔΔCT^ method. The primer sequences used for qRT-PCR are listed in Table S1.

### Flow cytometry analysis

Single-cell suspensions were obtained by dissociating the cells with TrypLE for 5 to 10 min at 37 °C. The cells were then fixed and permeabilized using the Transcription Factor Staining Buffer Set (Invitrogen, 00-5523-00) according to the manufacturer’s instructions. After staining with the specific antibodies listed in Table S2, the cells were passed through 40-μm cell strainers. Finally, the stained cells were analyzed using a FACSCelesta flow cytometer (BD, USA).

### Immunofluorescence staining

Organoids were collected at designated time points, washed with PBS, and fixed overnight at 4 °C in 4% paraformaldehyde (PFA). Following fixation, the organoids were permeabilized with 0.5% Triton X-100 for 20 min and blocked with goat or donkey serum for 60 min. After each step, the organoids were washed 3 times with PBS. The organoids were then incubated with primary antibodies diluted in PBS overnight at 4 °C, followed by incubation with secondary antibodies in PBS for 1 h at room temperature in the dark. Nuclei were counterstained with 4′,6-diamidino-2-phenylindole (DAPI; Beyotime Biotechnology, C1006) for 5 min. Immunostaining images were captured using a single-photon confocal microscope (Ti-E A1, Nikon, Japan). The antibodies used in this study are listed in Table S2. All immunofluorescence staining experiments were performed with at least 3 independent biological replicates, and representative images were taken.

### Western blot analysis

Cells were lysed on ice using radioimmunoprecipitation assay lysis buffer (Solarbio, R0020) supplemented with phenylmethylsulfonyl fluoride (Beyotime Biotechnology, ST505), a protease inhibitor (Beyotime Biotechnology, P1005), and a phosphatase inhibitor (Beyotime Biotechnology, P1081). Protein concentrations were determined using the BCA (bicinchoninic acid) Protein Assay Kit (Biosharp, BL521A) following the manufacturer’s instructions. Western blotting (WB) was performed using standard procedures. The antibodies used in this study are listed in Table S2.

### Analysis of liver function

All functional assays were performed using organoids cultured for 6 d in HCM. To analyze the secretion levels of albumin (ALB) and bile acids, media were collected at indicated time points and analyzed by using the Human ALB ELISA (enzyme-linked immunosorbent assay) Quantitation Kit (Bethyl, E88-129) and Total Bile Acid Detection Kit (NjjcBio, E003-2-1) according to the manufacturer’s instructions, respectively. To determine urea synthesis, 10 mM ammonium chloride (Sigma, A9434) was added to the culture medium and incubated for 24 h; then, the medium was collected, and the Urea Assay Kit (Solarbio, BC1535) was used for the quantification according to the manufacturer’s protocols. To analyze glycogen storge, the organoids were stained by the Periodic Acid-Schiff Staining Kit (Beyotime Biotechnology, C0142S) following the manufacturer’s manuals. For the uptake and excretion of indocyanine green (ICG), the organoids were cultured with 1 mg/ml of ICG (MedChemExpress, HY-D0711) for 1 h at 37 °C in 5% CO_2_, and ICG uptake by the organoids were captured under a microscope; then, the organoids were gently washed 3 times with PBS, and fresh medium was added. One hour after the incubation, the excretion of ICG from the organoids was examined under the microscope. For bile canaliculus assessment, the organoids were incubated with 2 μM CDFDA for 15 min at 37 °C; then, the organoids were rinsed with PBS 3 times, and fluorescence images were captured by single-photon confocal microscopy immediately. To assess cytochrome P450 (CYP) activity, organoids were incubated with the inducers of rifampicin (Sigma, R3501) at 25 μM or omeprazole (Sigma, O104) at 100 μM for inducing the expressions of CYPs for 48 h; then, CYP enzyme activities were measured by the P450-Glo Assay Kit (Promega, V9002, V8422, and V8791) according to the manufacturer’s instructions.

### Cell proliferation and fold-increase calculation

For the assessment of cell expansion, HB-orgs cultured with or without MG were dissociated into single cells and seeded at a density of 2 × 10^5^ cells per well into ultralow-attachment 6-well plates. Six days after the culture, viable cells were counted by trypan blue exclusion using a hemocytometer. The fold increase was calculated by the ratio of the total viable cell number at day 6 to the initial seeding number. This procedure was repeated for 4 consecutive passages, and results are presented as the mean ± standard deviation (SD) of 3 independent experiments.

### Cell cycle analysis

The cell cycle distribution was determined by the Cell Cycle Analysis Kit (Beyotime Biotechnology, C1052) and Apoptosis Analysis Kit (Beyotime Biotechnology, C1062M) according to the manufacturer’s manual. In brief, single-cell suspensions were fixed with 70% cold ethanol at 4 °C overnight. The next day, cells were washed with PBS and stained with a mixture of propidium iodide and RNase A. The cell cycle was detected by a BD FACSCelesta flow cytometer.

### Senescence-associated β-galactosidase staining

Senescence-associated β-galactosidase (SA-β-gal) staining was performed using the SA-β-gal Staining Kit (Beyotime Biotechnology, C0602) according to the manufacturer’s instructions. Briefly, the organoids were fixed with 4% PFA for 30 min at room temperature; then, the organoids were incubated in X-gal solution in the dark at 37 °C overnight. After staining, organoids were rinsed with PBS and randomly imaged by a phase-contrast microscope (Nikon, Japan).

### Apoptosis assay

The percentages of apoptotic cells in the organoids were evaluated by the Annexin V-FITC (fluorescein isothiocyanate) Apoptosis Kit (Beyotime Biotechnology, C1062M) according to the manufacturer’s protocol. Briefly, single-cell suspensions of the organoids were obtained by dissociation with TrypLE for 5 min; then, cells were stained with annexin V–FITC and propidium iodide for 20 min in the dark on ice. After the incubation, the percentages of apoptotic cells were analyzed by flow cytometry (BD FACSCelesta).

### Detection of reactive oxygen species

Intracellular reactive oxygen species (ROS) levels were detected by the Reactive Oxygen Species Assay Kit (NjjcBio, E004-1-1) according to the manufacturer’s manual. In brief, the organoids were incubated with serum-free medium supplemented with 5 μM 2′,7′-dichlorodihydrofluorescein diacetate (DCFH-DA) probe and cultured at 37 °C for 20 min. Then, the organoids were rinsed with PBS 3 times and measured by single-photon confocal microscopy. Alternatively, organoids were dissociated into single cells with TrypLE for 5 min and the percentage of ROS-positive cells was determined by flow cytometry (BD FACSCelesta).

### Assessment of lysosomal activity

The activity of lysosomes was assessed by Lyso-Tracker Red (Beyotime Biotechnology, C1046) according to the manufacturer’s manuals. In brief, the organoids were cultured in the medium supplemented with 50 nM Lyso-Tracker Red 37 °C for 1 h; then, the organoids were rinsed with PBS and fixed and permeabilized, and then nuclei were counterstained with DAPI. Finally, the fluorescence images were observed using single-photon confocal microscopy.

### Terminal deoxynucleotidyl transferase-mediated dUTP nick-end labeling assay

The terminal deoxynucleotidyl transferase-mediated dUTP nick-end labeling (TUNEL) assay was performed by using the one-step TUNEL Apoptosis Assay Kit (Beyotime Biotechnology, C1086) according to the manufacturer’s instructions. In brief, the organoids were fixed with 4% PFA for 30 min and permeabilized with 0.5% Triton X-100 for 20 min at room temperature and then incubated with the TUNEL detection mixture for 1 h in the dark. Finally, nuclei were counterstained with DAPI for 5 min. After staining, the organoids were rinsed with PBS and captured randomly by single-photon confocal microscopy.

### Transmission electron microscopy

The organoids were fixed by 2.5% glutaraldehyde at 4 °C overnight, postfixed in 1% osmium tetroxide for 2 h, then dehydrated in a graded series of ethanol, and embedded. Samples were sectioned by Leica UC7 and stained with 2% uranyl acetate for 20 min, followed by lead citrate for 12 min. Images were acquired on an FEI transmission electron microscope (Tecnai G2 Spirit, USA).

### Analysis of total RNA-seq

Total RNA from HB-orgs cultured with or without MG, P-hep-orgs, NP-hep-spheres, and PHHs were extracted using the RNAiso Plus kit according to the manufacturer’s instructions. The complementary DNA libraries for RNA-seq were generated using the NEBNext Ultra RNA Library Prep Kit for Illumina (NEB, E7530). Sequencing was performed by Novogene (Beijing, China) on an Illumina HiSeq X Ten sequencer with 150-bp paired-end sequencing reaction. Differentially expressed genes (DEGs) were analyzed by DESeq2 using counts. Genes with *P* ≤ 0.05 and |log_2_ fold change| ≥ 2 were identified as DEGs. Original data were uploaded to the Gene Expression Omnibus database (accession number: GSE239550).

### Statistical analysis

Sample sizes are indicated on the corresponding graph or figure legend; otherwise, *n* = 3, which represents the number of biological replicates that were analyzed in each experimental group. Data are expressed as the mean ± SD or standard error of the mean. Statistical analysis was performed using SPSS. The unpaired, 2-tailed Student *t* test and one-way analysis of variance (Tukey correction for multiple comparisons) were used to evaluate statistical significance. Differences were considered statistically significant at *P* value < 0.05.

## Results

### MG was required for the expansion of HB-orgs

**Fig. 1.**
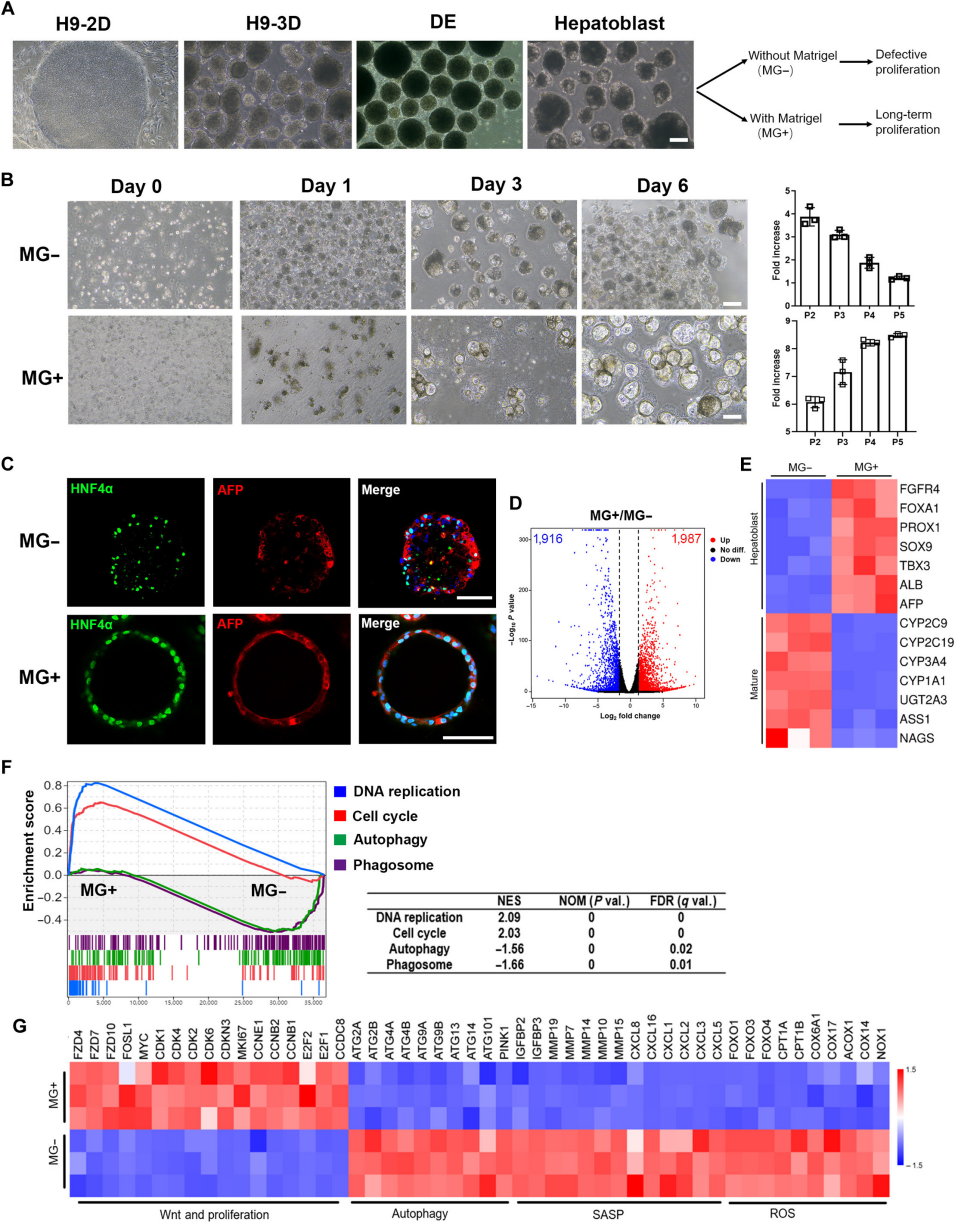
Comparison of hepatoblast organoids (HB-orgs) cultured with or without Matrigel. (A) Representative cell morphologies at different stages during the differentiation of hepatoblasts. Scale bar = 100 μm. (B) Representative morphologies and fold changes in viable cell numbers of HB-orgs cultured with 5% Matrigel (MG+) or without Matrigel (MG−). Fold change was calculated by the ratio of viable cell numbers at day 6 to the initial seeding cell number at day 0. Data represent 3 independent experiments (*n* = 3). Scale bar = 100 μm. (C) Representative immunofluorescence images for hepatic progenitor cell markers hepatocyte nuclear factor 4α (HNF4α) and α-fetoprotein (AFP) in HB-orgs cultured without Matrigel (MG−) or with 5% Matrigel (MG+) from 3 independent experiments are shown. Nuclei were stained with 4′,6-diamidino-2-phenylindole (DAPI). Scale bar = 100 μm. (D) Volcano plot of HB-orgs cultured with or without Matrigel. (E) Heatmap of HB-orgs cultured with or without Matrigel on hepatoblast- and mature-related genes. (F) Gene set enrichment analysis (GSEA) was performed to identify enriched pathways between HB-orgs cultured with or without Matrigel. (G) Heatmap of HB-orgs cultured with or without Matrigel on Wnt/proliferation-, autophagy-, senescence-associated secretory phenotype (SASP)-, and reactive oxygen species (ROS)-related genes. DE, definitive endoderm; NES, normalized enrichment score; NOM, nominal *P* value; FDR, false discovery rate.

The hESCs exhibited great proliferative capacity as aggregates in mTeSR1 medium under 3D suspension culture in our previous study [20]. In order to culture differentiated HBs as hESC aggregates in vitro, we developed a new differentiation method for the generation of HBs from H9 cells under 3D suspension culture system (Fig. 1A), as well as a defined HB medium containing chemicals and growth factors that supported HB proliferation [21,22]. Although H9 cells do not require an ECM to expand efficiently in the form of aggregates under 3D suspension culture conditions, ECM is known to support hepatocyte viability and function [23]; we decided to compare the effect of 5% (vol/vol) MG on the growth of differentiated HB spheres and found that cells from HB spheres reaggregated into small spheres and grew larger after being dissociated into single cells without MG, but the proliferation rate was decreased during passaging (Fig. 1B), although they retained the expression of the HB markers α-fetoprotein (AFP) and hepatocyte nuclear factor 4α (HNF4α) (Fig. 1C). In contrast, by adding 5% MG into the expansion system, HBs formed organoid structures (HB-orgs) and expanded efficiently through multiple passages, with an increase in cell numbers under the support of MG (Fig. 1B), and they highly expressed the HB markers AFP and HNF4α (Fig. 1C). These results indicated that 5% MG was essential for HB proliferation and organoid formation in our system.

### Characterization of HB-orgs cultured with or without MG

The interaction between cells and the ECM is crucial for regulating hepatocyte functions and proliferation [24], but the molecular mechanisms underlying the interaction between HB-orgs and the ECM remain unclear. To investigate this, total RNA-seq was performed on HB-orgs cultured with or without MG, with samples collected at day 6 after the culture to compare DEGs. The volcano plot revealed 1,987 up-regulated and 1,916 down-regulated genes in HB-orgs cultured with MG (|log_2_ fold change| ≥ 2, *P* ≤ 0.05) (Fig. 1D). The heatmap (Fig. 1E) shows that HB-orgs cultured with MG expressed higher levels of HB-related genes than mature hepatocyte markers, compared with those without MG, suggesting maintenance of an undifferentiated state. Kyoto Encyclopedia of Genes and Genomes (KEGG) and gene set enrichment analyses further showed that pathways related to cell proliferation, such as DNA replication, cell cycle, and Wnt signaling, were enhanced in MG-cultured HB-orgs, whereas pathways linked to autophagy, mitophagy, and cellular senescence were enriched in those cultured without MG (Fig. 1F and Fig. S1a).

**Fig. 2.**
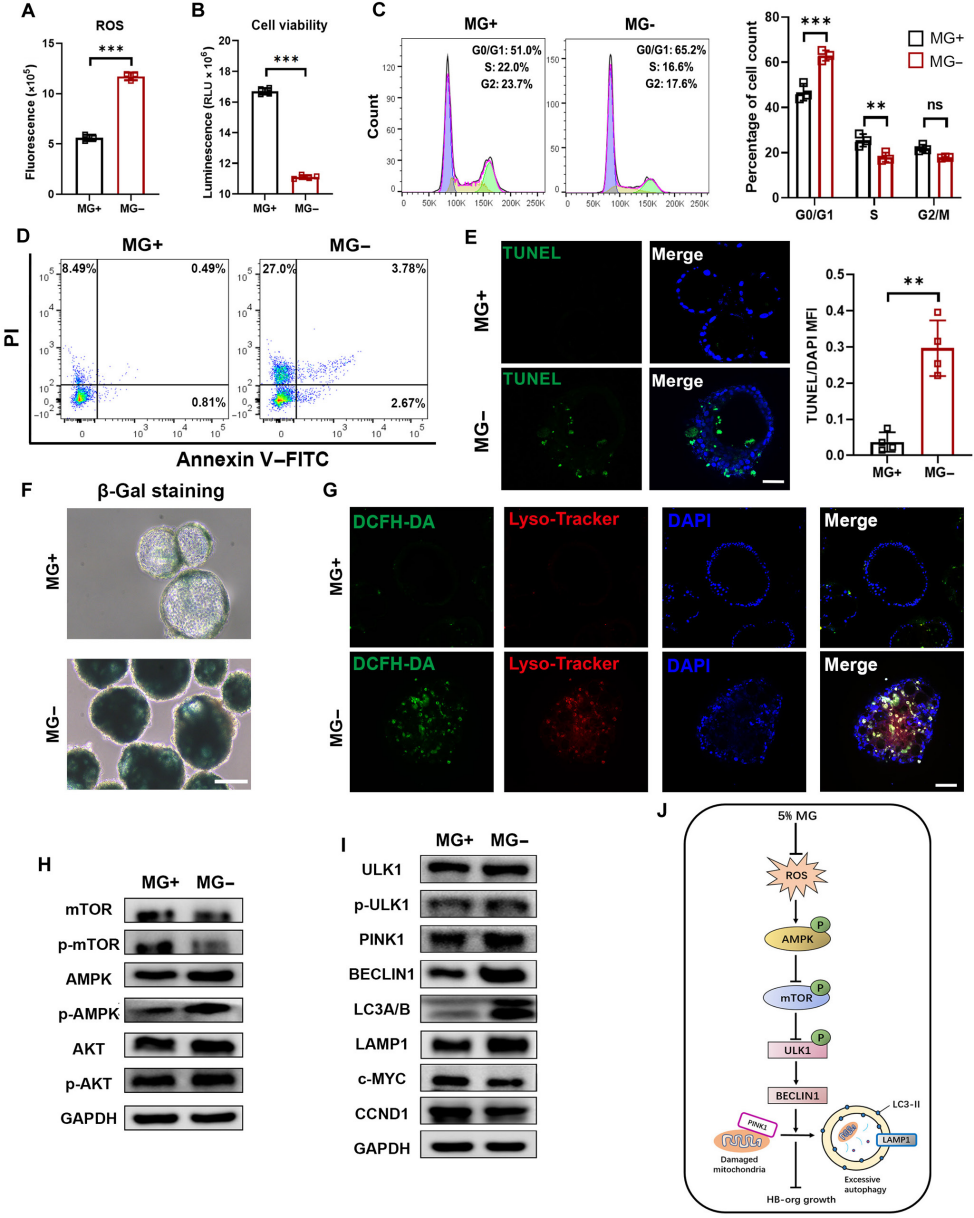
Characterizations of HB-orgs cultured with or without Matrigel. (A to D) Comparisons of ROS levels (*n* = 3 independent experiments) (A), cell viability (*n* = 4 independent experiments) (B), cell cycle distribution (*n* = 3 independent experiments) (C), and apoptotic cells (D) of HB-orgs cultured in MG+ and MG− conditions. (E) Cell apoptosis was measured by terminal deoxynucleotidyl transferase-mediated dUTP nick-end labeling (TUNEL) staining (*n* = 4 independent experiments). Nuclei were stained with DAPI. Scale bar = 50 μm. Quantification of TUNEL staining (right panel). (F) Representative images of β-galactosidase (β-Gal) staining of HB-orgs in MG+ and MG− conditions from 3 independent experiments. Scale bar = 100 μm. (G) Representative images of intracellular ROS and lysosome staining using 2′,7′-dichlorodihydrofluorescein diacetate (DCFH-DA) and Lyso-Tracker from 3 independent experiments. Nuclei were stained with DAPI. Scale bar = 100 μm. (H) Western blot analysis of autophagy-related signaling including mTOR, AMPK, and AKT. (I) Western blot analysis of autophagy-related markers including ULK1, p-ULK1, PINK1, BECLIN1, LC3A/B, LAMP1, and Wnt-signaling-related markers including c-MYC and CCND1 (I) of HB-orgs in MG+ and MG− conditions. Western blot analyses were performed in 3 independent experiments. (J) Schematic illustration of the regulation mechanism enabling the expansion of HB-orgs under 3-dimensional (3D) suspension conditions with Matrigel. Results are presented as mean ± SD. **P < 0.01 and ***P < 0.001. ns, not significant; PI, propidium iodide; FITC, fluorescein isothiocyanate; MFI, mean fluorescence intensity; GAPDH, glyceraldehyde-3-phosphate dehydrogenase; MG, Matrigel.

Autophagy, a self-degradative process, is crucial for energy balance and is typically considered a survival mechanism [25]. However, excessive autophagy can lead to cell cycle arrest and promote cellular senescence [26]. Given the up-regulation of autophagy, we hypothesized that excessive autophagy might explain why HB-orgs could not be maintained without MG. Consistent with this speculation, genes related to Wnt signaling and cell proliferation were elevated in MG-cultured HB-orgs (Fig. 1G and Fig. S1b), whereas many autophagy-related genes were up-regulated in those cultured without MG (Fig. 1G and Fig. S1c and d). Because ROS are key drivers of autophagy activation [26], we assessed ROS-related gene expressions and intracellular ROS levels using DCFH-DA probes. The results showed that both gene expression levels and ROS levels were higher in HB-orgs cultured without MG (Figs. 1G and 2A and Fig. S1e).

To further demonstrate the impact of excessive autophagy on HB-orgs, we found that HB-orgs cultured without MG showed a significant reduction in cell viability (Fig. 2B) and exhibited a higher percentage of cells whose cell cycle was arrested at the G0/G1 phase (Fig. 2C). Notably, a higher number of apoptotic cells at early (2.67% vs. 0.81%) and late stages (27.0% vs. 8.49%) was detected in HB-orgs without MG compared to those cultured with MG (Fig. 2D), and the TUNEL assay also demonstrated a higher apoptotic percentage in HB-orgs without MG (Fig. 2E). Additionally, many senescence-associated secretory phenotype genes [27] were up-regulated (Fig. 1G and Fig. S1f), and SA-β-gal-positive cells were increased prominently (Fig. 2F), indicating the occurrence of cell senescence, which likely contributed to the poor proliferation of HB-orgs cultured without MG. Increased cellular ROS and acidic lysosomes, markers of autophagy [28], were also observed in these HB-orgs using DCFH-DA and Lyso-Tracker staining (Fig. 2G).

KEGG pathway enrichment analysis revealed that AMPK signaling was activated in HB-orgs cultured without MG (Fig. S1a). WB analysis exhibited that p-AMPK, but not p-AKT, was increased, and p-mTOR was reduced, indicating autophagy activation via the classical AMPK–mTOR pathway (Fig. 2H and Fig. S1g) [29]. Additionally, autophagy-related proteins (p-ULK1^Ser555^, BECLIN1, LC3A/B, and LAMP1) and mitophagy protein PINK1 were significantly up-regulated, whereas proliferation-related proteins c-MYC and CCND1 were down-regulated (Fig. 2I and Fig. S1h), indicating the occurrence of enhanced autophagy and reduced proliferation. Taken together, these results demonstrate that HB-orgs cultured in MG maintained ROS homeostasis, further inhibited AMPK–mTOR signaling, and avoided excessive autophagy, thereby retaining active proliferative capacity for long-term culture under 3D suspension conditions (Fig. 2J).

### MG was required for the polarization of P-hep-orgs

**Fig. 3.**
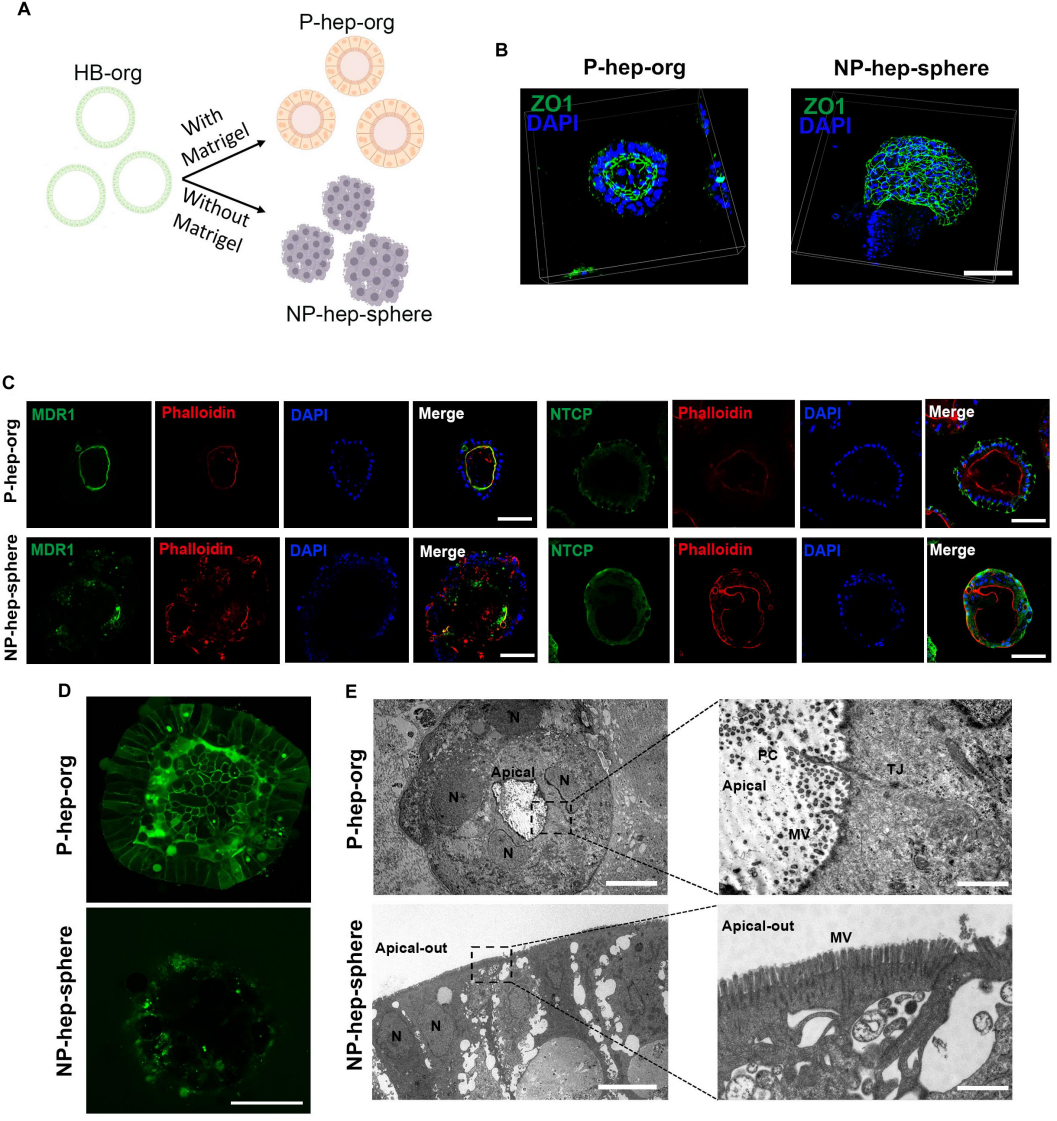
Characterization of hepatocyte polarization between polarized hepatocyte organoids (P-hep-orgs) and nonpolarized hepatocyte spheres (NP-hep-spheres). (A) Schematic illustration of differentiating HB-orgs into P-hep-orgs and NP-hep-spheres with or without Matrigel. (B) Representative 3D confocal immunofluorescence (IF) images of zonula occludens-1 (ZO1) from 3 independent experiments. Scale bar = 100 μm. (C) Representative IF images for polarity markers MDR1, NTCP, and F-actin (phalloidin staining) from 3 independent experiments. Nuclei were stained with DAPI. Scale bar = 100 μm. (D) Representative images of bile canaliculus staining using 5(6)-carboxy-2′,7′-dichlorofluorescein diacetate (CDFDA) from 3 independent experiments. Scale bar = 100 μm. (E) Representative images of transmission electron microscopy (TEM) from 3 independent experiments. N, nucleus; TJ, tight junction; MV, microvilli; PC, primary cilium. Scale bars = 10 μm (left), 2 μm (top right), and 1 μm (bottom right).

Hepatocyte polarization is essential for bile synthesis, secretion, and the exchange of components with sinusoidal blood [30]. Moreover, hepatocyte polarization is influenced by cell–ECM interactions, as seen in sandwich and transwell models [31]. To investigate the influence of MG on the maturation of HB-orgs, we compared the structures of hepatocyte organoids differentiated from HB-orgs cultured with or without MG (Fig. 3A). Six days after differentiation, we found that adding 5% MG enabled the formation of well-organized polarized structures in hepatocyte organoids, termed P-hep-orgs. In contrast, hepatocytes differentiated without MG formed NP-hep-spheres. Zonula occludens-1 (ZO1), a tight junction marker of polarized hepatocytes, located in the inner face of P-hep-orgs, demonstrated a typically polarized, columnar epithelial organization, while ZO1 was disorderly in NP-hep-spheres (Fig. 3B). Furthermore, we assessed hepatocyte polarization markers by immunostaining. MDR1, an apical marker, co-localized with F-actin (phalloidin) at the inner (apical) surface of P-hep-orgs, whereas NTCP, a basolateral marker, localized to the outer (basal) surface. Both markers were disorganized in NP-hep-spheres, similar to ZO1 (Fig. 3C). Moreover, bile-canaliculus-like structures were generated and well organized in P-hep-orgs and were clearly visualized by CDFDA staining; by contrast, NP-hep-spheres displayed dispersed, reduced, and irregular CDFDA staining (Fig. 3D). Through the observation by a transmission electron microscope, we also found the representative apical microvilli, tight junction, and primary cilium in apical lumina in P-hep-orgs, whereas NP-hep-spheres showed only external microvilli, resembling apical-out organoids (Fig. 3E) [32]. Additionally, a large number of hepatocyte-polarization-associated genes were highly expressed in P-hep-orgs (Fig. S2a), confirming their polarized state. Overall, these results demonstrate that MG was necessary for the polarization of P-hep-orgs in our 3D suspension culture system.

### MG was required for P-hep-orgs to exhibit hepatocyte functions

**Fig. 4.**
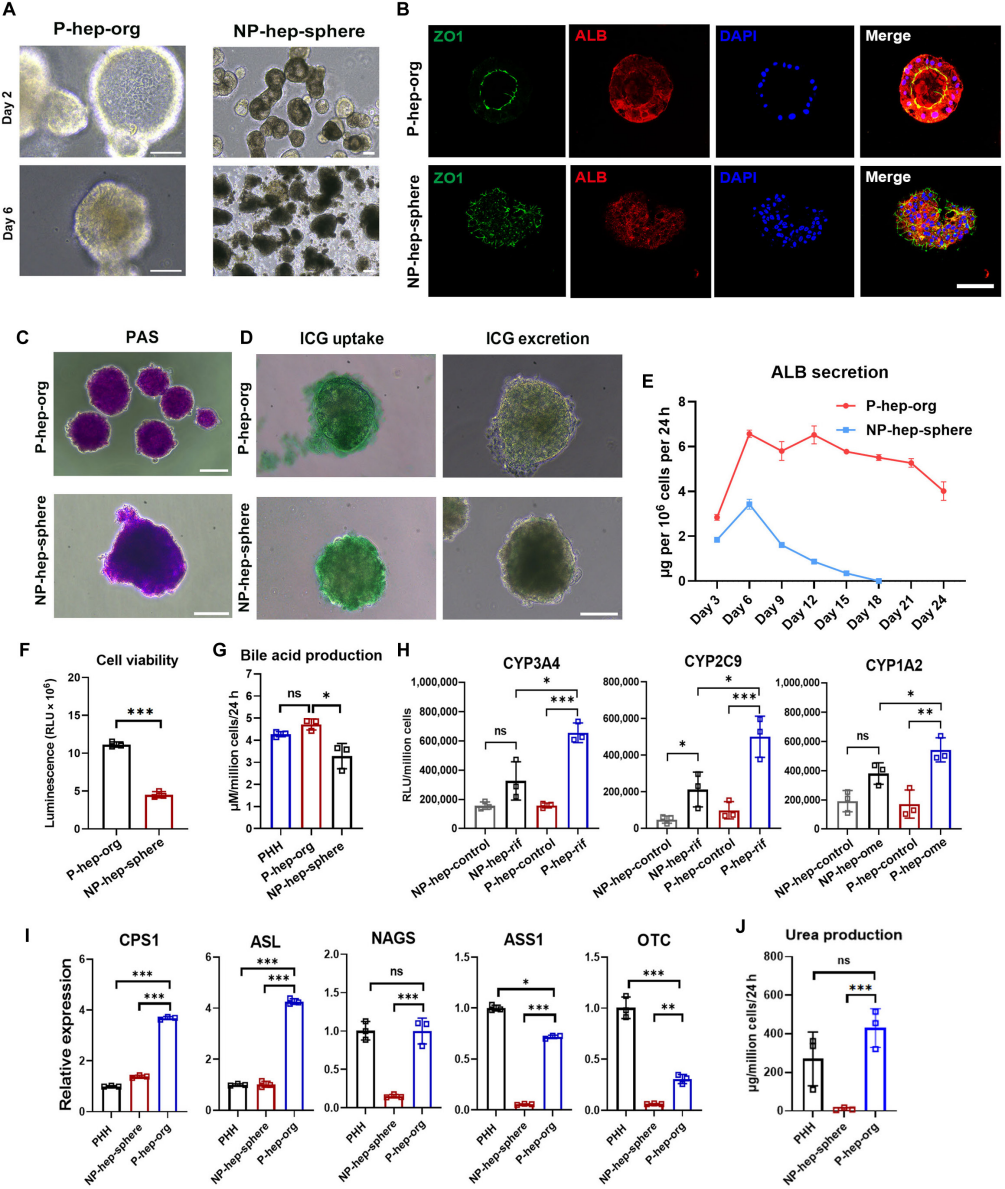
Characterization of hepatocyte functions between P-hep-orgs and NP-hep-spheres. (A) Representative morphology images of indicated groups at days 2 and 6 during the differentiation from 3 independent experiments. Scale bar = 50 μm for P-hep-orgs and 100 μm for NP-hep-spheres. (B) Representative IF images for mature hepatocyte markers in P-hep-orgs or NP-hep-spheres from 3 independent experiments. Nuclei were stained with DAPI. Scale bar = 100 μm. (C and D) Representative images of periodic acid–Schiff (PAS) staining (C) and indocyanine green (ICG) uptake and excretion (D) of the indicated groups from 3 independent experiments. Scale bar = 100 μm. (E) Albumin (ALB) secretions by P-hep-orgs and NP-hep-spheres at different time points during the differentiation (*n* = 3 independent experiments). (F) Assessments of cell viability in the indicated groups at day 12 (*n* = 3 independent experiments). (G) Assessments of bile acid production in the indicated groups at day 6 (*n* = 3 independent experiments). (H) Cytochrome P450 (CYP) activities in response to inducers rifampicin (rif) or omeprazole (ome) in the indicated groups at day 6 (*n* = 3 independent experiments). (I) The relative expression levels of urea-cycle-related genes were analyzed by quantitative reverse transcription polymerase chain reaction (qRT-PCR) in the indicated groups at day 6 (*n* = 3 independent experiments). (J) Assessment of urea production in the indicated groups at day 6 (*n* = 3 independent experiments). Results are presented as mean ± SD. *P < 0.05, **P < 0.01, and ***P < 0.001. PHH, primary human hepatocyte.

Hepatocyte polarization is crucial for hepatocyte function, even in vitro. P-hep-orgs displayed an organized structure with a typical polygonal shape (Fig. 4A) and the expression of mature hepatocyte markers ALB, α1AT, and ASGPR (Fig. 4B and Fig. S2b), whereas NP-hep-spheres showed a disorganized arrangement with decreased expression of these mature markers (Fig. 4B and Fig. S2b). Functional assessments showed that both P-hep-orgs and NP-hep-spheres could store glycogen (periodic acid–Schiff staining) and take up and excrete ICG (Fig. 4C and D). However, P-hep-orgs exhibited significantly higher ALB secretion that persisted for over 21 d, whereas the secretion of ALB declined in NP-hep-spheres after 6 d (Fig. 4E), concomitant with significant cell death and decreased cell viability measured at day 12 (Fig. 4A and F). Moreover, 6 d after the differentiation, P-hep-orgs showed higher bile acid production (Fig. 4G) and greater activities of CYP enzymes such as CYP3A4, CYP2C9, and CYP1A2 upon exposure to the inducers rifampicin and omeprazole, compared to NP-hep-spheres (Fig. 4H). Urea-synthesis-related genes were also expressed at levels similar to or higher than those of PHHs (Fig. 4I). In particular, 24 h after treatment with 10 mM ammonium chloride, P-hep-orgs produced significantly more urea than NP-hep-spheres did, with levels comparable to that of PHHs (Fig. 4J), highlighting their strong ammonia-elimination capacity. These results indicate that P-hep-orgs exhibited superior hepatocyte functions compared with NP-hep-spheres in protein synthesis, drug metabolism, and urea metabolism.

### In vitro toxicological screening using P-hep-orgs

**Fig. 5.**
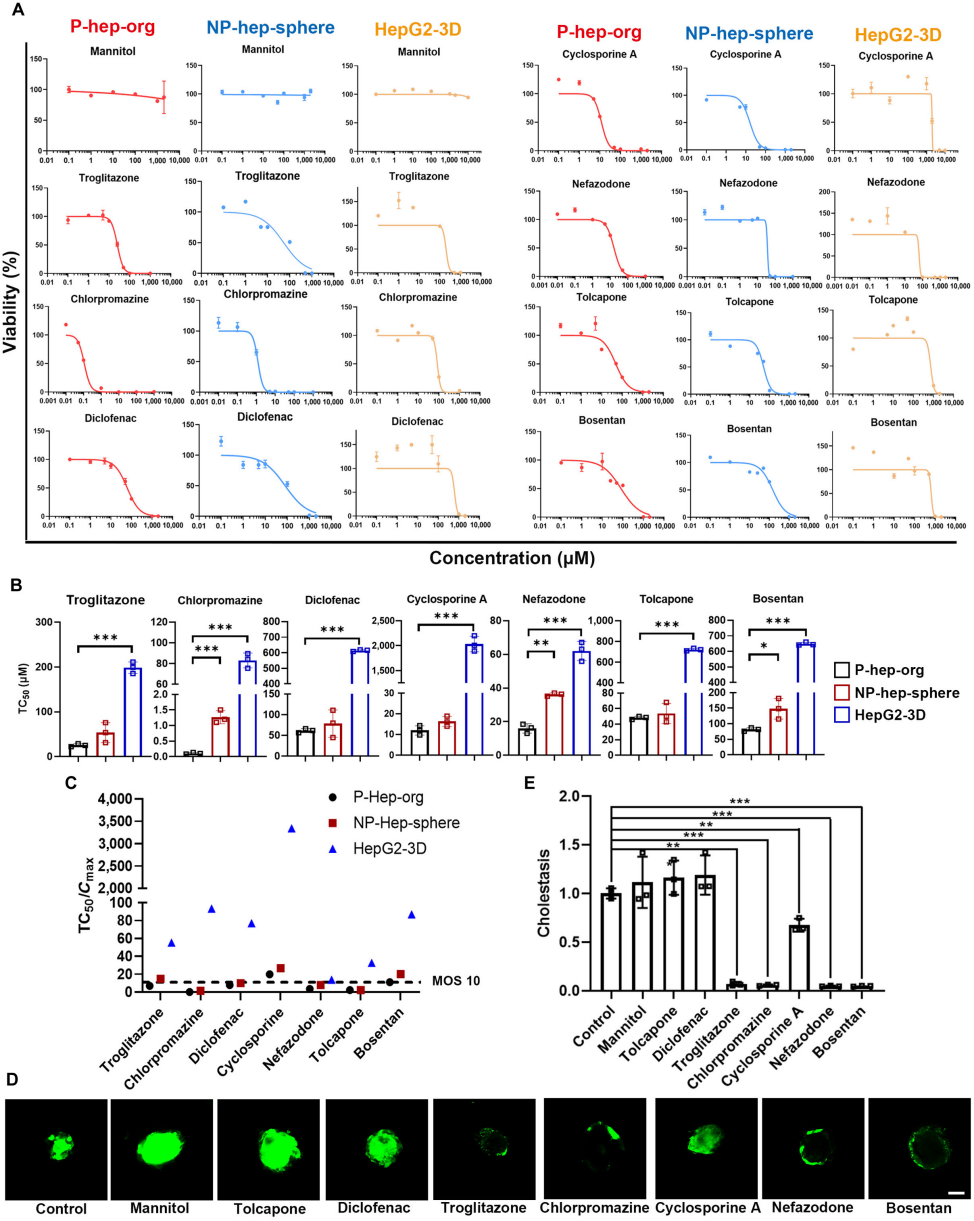
Detection of drug responses and cholestasis using hepatotoxic compounds. (A) Dose-dependent toxicity curves of different compounds showing cell viabilities among P-hep-orgs, NP-hep-spheres, and HepG2-3D 48 h after the treatment (*n* = 3 independent experiments). (B) TC_50_ values of each indicated group 48 h after treatments with different compounds (*n* = 3 independent experiments). (C) Summary of margin of safety (MOS) values from in vitro toxicity tests. The dashed line indicates MOS at 10. (D and E) Representative fluorescence images of CDFDA transportation in P-hep-orgs posttreatment with specific drugs (D) and the quantification of fluorescence intensity (*n* = 3 independent experiments) (E). Scale bar = 100 μm. Results are presented as mean ± SD. *P < 0.05, **P < 0.01, and ***P < 0.001.

A growing body of evidence suggests that the use of inappropriate cell models to predict human hepatotoxicity is a major factor behind numerous drug withdrawals [33]. Traditionally, human liver cell lines, such as HepG2 cells cultured under 2D conditions, have been used for prediction toxicity [34]. However, their immaturity and unsuitable culture conditions limit their relevance to human liver pathophysiology [35]. Given that our functional P-hep-orgs were polarized and cultured in 3D systems, which could better mimic liver characteristics, we sought to assess their potential for predicting hepatotoxicity. We tested 7 known hepatotoxic compounds, along with mannitol (a compound considered safe for hepatocytes), on P-hep-orgs, NP-hep-spheres, and HepG2 cells, which were cultured in 3D suspension conditions (HepG2-3D). Since drug-induced liver injury can be caused by these compounds, cells were cultured in ultralow-attachment 96-well plates, exposed to the drugs for 48 h, and the viability of treated cells was measured using the CellTiter-Glo 3D assay. Results were summarized as dose-dependent toxicity curves and as TC_50_ values, defined as the concentration that reduced cell viability by 50%. These curves displayed a classic sigmoidal response of toxic metabolites, indicating that most drugs showed concentration-dependent cytotoxicity (Fig. 5A). Notably, the dose–responses varied across cell types. HepG2-3D exhibited the weakest drug response, with the highest TC_50_ values for all tested compounds (Fig. 5B and Table S3). In contrast, P-hep-orgs showed the highest drug sensitivity, with the lowest TC_50_ values across all compounds, outperforming both NP-hep-spheres and HepG2-3D (Fig. 5B and Table S3). We employed the margin of safety (MOS) approach where the ratio of TC_50_/*C*_max_ is used to evaluate the relevance of a test compound in a clinical setting; a lower MOS usually indicates that the in vitro toxic concentration is close to clinical exposure [36]. The MOS values obtained from P-hep-orgs were mostly within the range of 2 to 20 (Fig. 5C and Table S4), closely aligning with the clinically observed safety margins of the corresponding drugs. This finding demonstrates that P-hep-orgs provide a physiologically relevant platform that more accurately reflects human hepatic sensitivity to drug-induced toxicity, thereby serving as a reliable predictor of clinical risk. In contrast, HepG2-3D models yielded MOS values predominantly above 100, in some cases exceeding 3,000, substantially overestimating the safety margin and failing to reflect clinical outcomes (Fig. 5C and Table S4). Collectively, these results underscore that P-hep-orgs offer a more accurate and clinically relevant assessment of hepatotoxic risk than HepG2-3D, reinforcing their value as a predictive in vitro model for drug safety evaluation.

To confirm the responses of P-hep-orgs to hepatotoxic compounds, we evaluated cholestasis by treating them with various compounds at TC_25_ concentrations. Because the hydrolytic fluorescent product CDF of CDFDA would be transported into bile canaliculi primarily through the apical protein MRP2 [37], we used CDFDA staining to corroborate the occurrence of the cholestasis. Compared to controls, significant reductions in CDF fluorescence were observed in P-hep-orgs treated with troglitazone, chlorpromazine, cyclosporine A, nefazodone, and bosentan (Fig. 5D and E), indicating the occurrence of cholestasis, consistent with reported drug effects [38]. These results highlighted P-hep-orgs as an effective human hepatocyte model for predicting hepatotoxicity and responses during drug development.

### Comparison of transcriptome profiles between P-hep-orgs and NP-hep-spheres

**Fig. 6.**
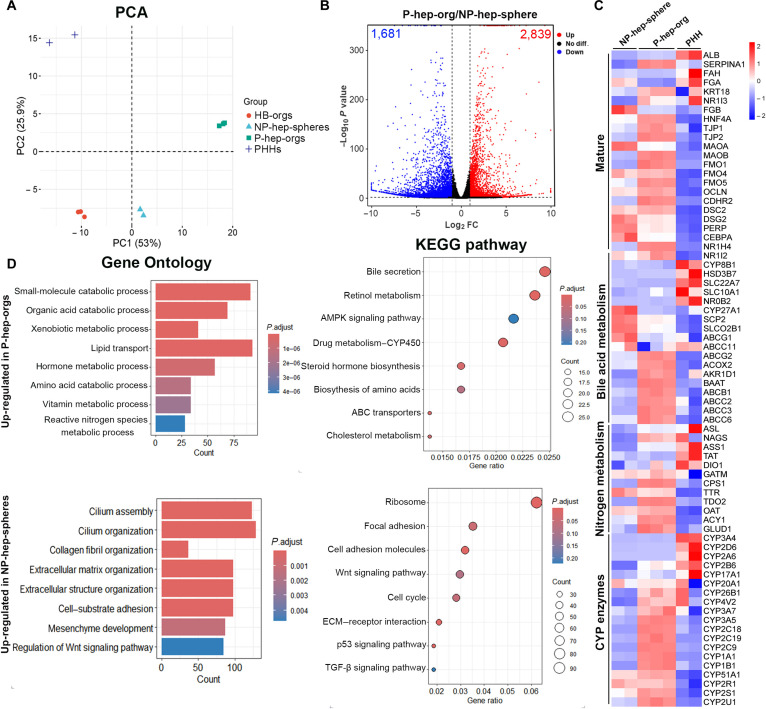
RNA sequencing (RNA-seq) analysis between P-hep-orgs and NP-hep-spheres. (A) Principal component analysis (PCA) of HB-orgs, NP-hep-spheres, P-hep-orgs, and PHHs. (B) Volcano plot of P-hep-orgs and NP-hep-spheres. (C) Heatmap of P-hep-orgs, NP-hep-spheres, and PHHs for genes related to mature hepatocytes, bile acid metabolism, nitrogen metabolism, and CYP enzymes. (D) Analysis of Gene Ontology and Kyoto Encyclopedia of Genes and Genomes (KEGG) pathway between P-hep-orgs and NP-hep-spheres. FC, fold change; ECM, extracellular matrix.

To further compare differences between P-hep-orgs and NP-hep-spheres, RNA-seq analysis was performed. Principal component analysis revealed clear separation between P-hep-orgs and NP-hep-spheres: P-hep-orgs clustered closer to PHHs, whereas NP-hep-spheres clustered HB-orgs, suggesting that P-hep-orgs were in a more mature state and NP-hep-spheres remained immature (Fig. 6A). Compared with NP-hep-spheres, 2,839 genes were up-regulated and 1,681 genes were down-regulated in P-hep-orgs (Fig. 6B). The up-regulated genes in P-hep-orgs were associated with mature hepatocyte functions, including bile acid metabolism (*ABCG2*, *ACOX2*, and *ABCB1*), nitrogen metabolism (*ASL*, *NAGS*, and *ASS1*), drug metabolism (*CYP1A1*, *CYP2C9*, *CYP2C19*, *UGT1A3*, and *UGT2B4*), lipid metabolism (*APOA1*, *APOC3*, and *APOB*), and glucose metabolism (*GYS2*, *FBP1*, and *PPP2CB*) (Fig. 6C and Fig. S3). These results demonstrated that P-hep-orgs expressed higher levels of genes related to hepatocyte functions.

To further investigate functional roles of the up-regulated genes in P-hep-orgs and NP-hep-spheres, Gene Ontology (GO) and KEGG pathway analyses were performed. GO analysis indicated that the up-regulated genes in P-hep-orgs were enriched in metabolic and catabolic processes, including nitrogen, vitamin, amino acid, lipid, organic acid, and hormone metabolism. In contrast, the up-regulated genes in NP-hep-spheres were primarily involved in ECM organization and cilium assembly (Fig. 6D), suggesting that P-hep-orgs exhibited a more pronounced expression of genes related to liver cell functions. KEGG analysis confirmed that the up-regulated genes in P-hep-orgs were enriched in metabolism-related pathways, including drug metabolism (Fig. 6D). Additionally, pathways such as AMPK signaling, bile secretion, and ABC transporters were enriched (Fig. 6D); these pathways have been reported to support hepatocyte polarization in sandwich hepatocyte models [39]. By contrast, NP-hep-spheres showed enrichment in pathways related to cell cycle, Wnt signaling, and TGF-β signaling (Fig. 6D), highlighting their immature state.

The ECM is crucial for cell polarization [40], yet the mechanism underlying hepatocyte polarization in 3D suspension culture remains unclear. In the absence of MG support, NP-hep-spheres expended more energy in organizing the ECM to create a more suitable growth microenvironment, as indicated by GO analysis (Fig. 6D). This, in turn, results in an unpolarized structure and an immature state. Therefore, polarization in P-hep-orgs under a 3D suspension culture system is likely driven by the interaction among MG (ECM), integrins, and AMPK signaling.

### The polarization mechanism of P-hep-orgs

**Fig. 7.**
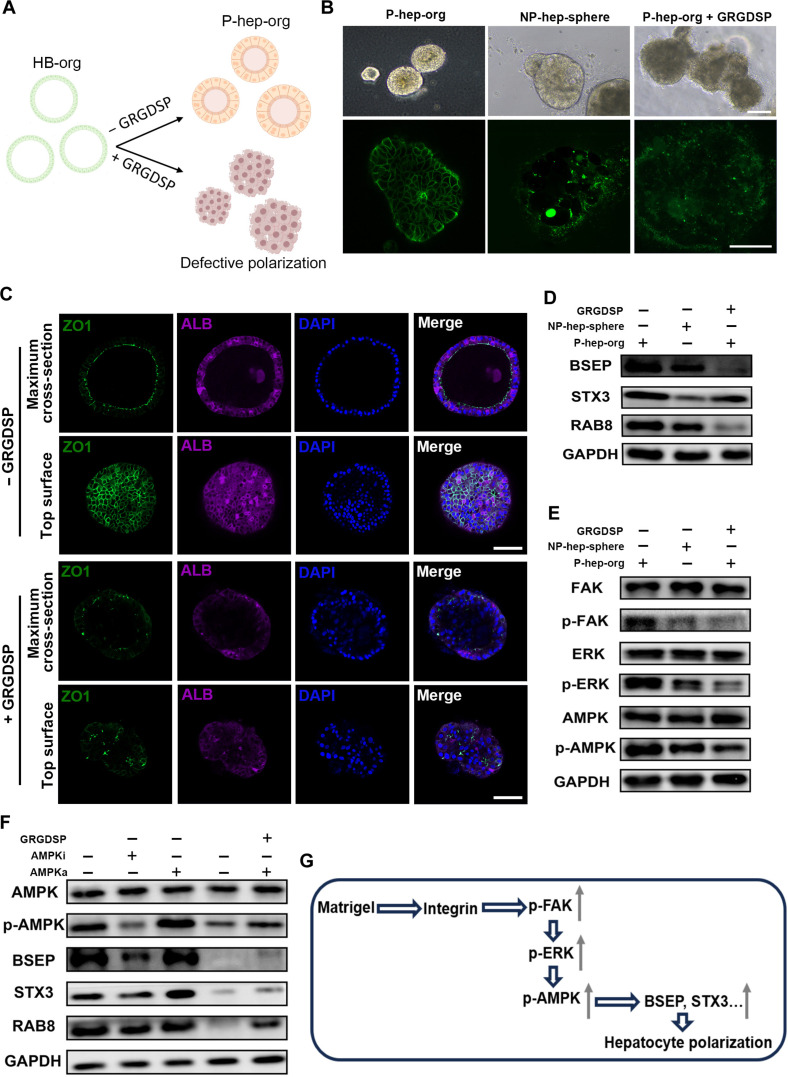
The polarization mechanism of P-hep-orgs under 3D suspension conditions with the support of Matrigel. (A) Schematic illustration of hepatocyte differentiation from HB-orgs without or with the integrin inhibitor GRGDSP. (B) Representative images of morphologies (top panel) and bile canaliculus staining with CDFDA (bottom panel) in P-hep-org, NP-hep-sphere, or P-hep-org treated with 100 μM GRGDSP from 3 independent experiments. Scale bar = 100 μm for both panels. (C) Representative IF images for ALB and ZO1 in P-hep-org treated with or without GRGDSP from 3 independent experiments. Nuclei were stained with DAPI. Scale bar = 100 μm. (D and E) Western blot analysis of polarization-related proteins, including bile salt export pump (BSEP), syntaxin 3 (STX3), and Ras-related protein Rab-8 (RAB8). (D) and the activation of polarization-pathway-related proteins pFAK, pERK, and pAMPK (E) among different cells. (F) Western blot analysis of polarization-related protein expressions of AMPK, p-AMPK, BSEP, STX3, and RAB8 after integrin–AMPK signaling modulation. Western blot analyses were performed in 3 independent experiments. (G) The schematic diagram of the proposed molecular mechanism for P-hep-org polarization under 3D suspension conditions with the support of Matrigel.

To test this hypothesis, we used the integrin-binding inhibitor Gly-Arg-Gly-Asp-Ser-Pro peptide (GRGDSP) to block the integrin interaction in P-hep-orgs (Fig. 7A). After treatment, CDFDA staining showed that P-hep-orgs lost their typical polarized morphology and resembled NP-hep-spheres (Fig. 7B). Furthermore, GRGDSP treatment markedly inhibited the formation/localization of the tight junction protein ZO-1 and partially reduced ALB expression (Fig. 7C), demonstrating that integrin blockade impairs hepatocyte polarization and maturation. Additionally, GRGDSP also decreased the expression of the polarization-related proteins bile salt export pump (BSEP), syntaxin 3 (STX3), and Ras-related protein Rab-8 (RAB8) (Fig. 7D and Fig. S4a). WB analysis further showed reduced levels of pFAK, pERK, and pAMPK (Fig. 7E and Fig. S4b), indicating that disruption of integrin signaling impaired the polarization of P-hep-orgs via the integrin-mediated FAK–ERK–AMPK pathway [39].

To confirm the role of AMPK signaling, we treated P-hep-orgs with an AMPK activator (AMPKa and AICAR) or inhibitor (AMPKi and dorsomorphin), with or without GRGDSP. Inhibition of AMPK signaling significantly down-regulated the polarization-associated proteins BSEP, STX3, and RAB8 in P-hep-orgs (Fig. 7F and Fig. S4c), while the activation on AMPK signaling partially restored the expression of the aforementioned proteins after integrin signaling was blocked (Fig. 7F and Fig. S4c). Additionally, inhibiting bile acid synthesis with the LXR inhibitor (SR9242) [41] down-regulated bile-acid-synthesis-related genes (*CYP7A1*, *CYP8B1*, and *LXR*) but did not affect the expression of bile acid excretion genes or levels of polarization proteins (Fig. S4d to g). These results indicated that bile acid production was not critical for polarization under our conditions. Overall, these results demonstrated that MG mediated the polarization in P-hep-orgs via integrin–FAK–ERK–AMPK signaling under 3D suspension culture (Fig. 7G).

## Discussion

In this study, we examined the crucial role of MG in the expansion of hESC-derived HB-orgs and in the polarization and function of mature P-hep-orgs differentiated from HB-orgs under 3D suspension culture conditions. Our results provide evidence that MG is not only necessary for the efficient expansion of HB-orgs but also plays a pivotal role in maintaining hepatocyte functions and promoting hepatocyte polarization under 3D suspension culture. Additionally, transcriptome analysis revealed higher expression of maturation-related genes in P-hep-orgs compared with those in NP-hep-spheres. Furthermore, the urea production by our P-hep-orgs was comparable to that of PHHs, indicating a strong capacity for ammonia metabolism and a suitable cell source for modeling/studying conditions associated with ammonia toxicity, including hepatic encephalopathy. CYP3A4, a major phase I drug-metabolizing enzyme, together with other important phase I enzymes (CYP1A2 and CYP2C9), which are responsible for the metabolism of most drugs, was also highly expressed in P-hep-orgs, indicating that P-hep-orgs constitute a valuable cell resource for pharmacological and toxicological studies.

While culture protocols for hepatocyte organoids have been extensively established, our understanding of how the ECM regulates hepatocyte organoids remains limited. In previous studies, we presented that hESCs could proliferate efficiently as uniform aggregates under 3D suspension culture conditions as a simple and convenient culture mode that supports high-yield expansion [20,42] and is compatible with various culture containers, such as horizontal or vertical stirred flasks [43,44], nonstirred flasks, and nutrient bag culture systems [45]. However, hepatocytes and HBs do not adapt well to the aggregate culture system, as we found that the ECM was indispensable for their growth, structural organization, and function in vitro. In addition, decellularized scaffold hydrogels derived from mammalian organs have been widely shown to support hepatocyte organoid culture. For instance, an ECM hydrogel derived from decellularized porcine small intestines could maintain liver organoids [46], and a human liver decellularized scaffold hydrogel supported the growth and differentiation of cholangiocyte organoids [47]. These studies demonstrated that a natural ECM-based microenvironment have great potential for hepatocyte organoid culture. Nevertheless, the mechanistic details of how the ECM regulates the growth, polarization, and function of hepatocyte organoids in 3D suspension remain poorly understood.

By comparing differentiated hepatocyte organoids cultured with or without MG using RNA-seq analysis, our present study demonstrated that the ECM in the microenvironment supported HB-orgs in maintaining ROS–autophagy homeostasis and facilitates long-term expansion of HB-orgs. Additionally, we found that the genes enriched in the AMPK signaling pathway were markedly up-regulated in P-hep-orgs. Given that AMPK activation has been directly linked to hepatocyte polarization in previous studies, we considered this pathway to be functionally important in our model. Moreover, the key variable between P-hep-orgs and NP-hep-spheres was the presence of MG, which provides an ECM microenvironment that facilitates cell–ECM communication and activates integrin signaling. Integrin engagement is known to activate canonical downstream effectors, including FAK and ERK [48], which can subsequently trigger AMPK [49], ultimately promoting hepatocyte polarization. Based on these transcriptomic analysis and established literature, we proposed that the integrin–FAK–ERK–AMPK axis was the most plausible signaling cascade explaining our observations under the current culture conditions. We acknowledge, however, that other integrin-mediated pathways may also contribute; for example, integrin–YAP signaling has been implicated in hepatocyte polarization [50], and our previous work demonstrated that YAP activation could promote hepatocyte polarity in 2D transwell culture [10]. However, our RNA-seq data did not provide direct evidence of YAP activation in 3D P-hep-orgs; thus, further studies will be needed to explore the potential contribution of integrin–YAP and other integrin-mediated pathways.

Our findings provide several insights that may guide the future generation of functional hepatocyte organoids. First, the essential role of MG in supporting both the expansion of HB-orgs and the polarization of mature P-hep-orgs highlights the importance of carefully optimizing ECM composition to maintain hepatocyte architecture and metabolic competence. Furthermore, 5% (vol/vol) MG is sufficient to support effective cell–ECM interactions in both HB-orgs and P-hep-orgs, which may facilitate the large-scale production of functional hepatocyte organoids for drug screening and potential clinical applications.

Although our findings support the involvement of integrin signaling in promoting the maturation and functional enhancement of P-hep-orgs, the precise integrin subunits mediating this effect remain undetermined. Evaluating more defined matrices, such as collagen, gelatin, or vitronectin, to determine whether they similarly enhance P-hep-org maturation via integrin signaling would be an important step toward identifying the responsible integrin heterodimers and guiding the rational design of synthetic hydrogels. Future studies employing subunit-specific knockdown, blocking antibodies, or affinity-based ligand assays, possibly combined with systematic comparisons of natural and synthetic matrices, will be necessary to pinpoint key integrin–ECM interactions underlying P-hep-org maturation. Addressing these questions will advance our understanding of how cell–matrix interactions regulate hepatocyte organoid development and provide a solid foundation for designing defined hydrogel systems for translational applications.

## Conclusion

In conclusion, our study provides strong evidence that MG plays critical roles in the expansion of HB-orgs through inhibition of ROS–AMPK–mTOR-mediated excessive autophagy to maintain ROS–autophagy homeostasis and in the polarization and function of mature P-hep-orgs via integrin-mediated signaling pathways, particularly the FAK–ERK–AMPK axis, under 3D suspension culture. These findings deepen our understanding of the roles of the ECM in hepatocyte organoids under 3D suspension culture and open new avenues for developing more pathophysiologically relevant models for liver disease modeling, drug testing, and regenerative medicine.

## Ethical Approval

The use of PHHs isolated from patients was approved by the Research Ethics Committee of Guangzhou First People’s Hospital (Ethical Approval No.: K-2019-167-02).

## Data Availability

The data that support the findings of this study are available from the corresponding author upon the request. RNA-seq datasets have been deposited in Gene Expression Omnibus (GEO) with accession number GSE239550.

## References

[1] Trefts E, Gannon M, Wasserman DH. The liver. Curr Biol. 2017;27(21):R1147–R1151. 29112863 10.1016/j.cub.2017.09.019 PMC5897118

[2] Hu H, Gehart H, Artegiani B, LÖpez-Iglesias C, Dekkers F, Basak O, van Es J, de Sousa Lopes SM, Begthel H, Korving J, et al. Long-term expansion of functional mouse and human hepatocytes as 3D organoids. Cell. 2018;175(6):1591–1606.e19. 30500538 10.1016/j.cell.2018.11.013

[3] Huch M, Gehart H, Van Boxtel R, Hamer K, Blokzijl F, Verstegen MM, Ellis E, Van Wenum M, Fuchs SA, De Ligt J, et al. Long-term culture of genome-stable bipotent stem cells from adult human liver. Cell. 2015;160(1–2):299–312. 25533785 10.1016/j.cell.2014.11.050 PMC4313365

[4] Wu F, Wu DI, Ren Y, Huang Y, Feng BO, Zhao N, Zhang T, Chen X, Chen S, Xu A. Generation of hepatobiliary organoids from human induced pluripotent stem cells. J Hepatol. 2019;70(6):1145–1158. 30630011 10.1016/j.jhep.2018.12.028

[5] Chhabra A, Song HH, Grzelak KA, Polacheck WJ, Fleming HE, Chen CS, Bhatia SN. A vascularized model of the human liver mimics regenerative responses. Proc Natl Acad Sci USA. 2022;119(28): Article e2115867119. 35763565 10.1073/pnas.2115867119 PMC9282349

[6] Peng WC, Kraaier LJ, Kluiver TA. Hepatocyte organoids and cell transplantation: What the future holds. Exp Mol Med. 2021;53(10):1512–1528. 34663941 10.1038/s12276-021-00579-x PMC8568948

[7] Gissen P, Arias IM. Structural and functional hepatocyte polarity and liver disease. J Hepatol. 2015;63(4):1023–1037. 26116792 10.1016/j.jhep.2015.06.015 PMC4582071

[8] Blau BJ, Miki T. The role of cellular interactions in the induction of hepatocyte polarity and functional maturation in stem cell-derived hepatic cells. Differentiation. 2019;106:42–48. 30878880 10.1016/j.diff.2019.02.006

[9] Cohen D, Brennwald PJ, Rodriguez-Boulan E, Müsch A. Mammalian PAR-1 determines epithelial lumen polarity by organizing the microtubule cytoskeleton. J Cell Biol. 2004;164(5):717–727. 14981097 10.1083/jcb.200308104 PMC2172160

[10] Wang J, Situ P, Chen S, Wu H, Zhang X, Liu S, Wang Y, Xie J, Chen H, Duan Y. Hepatic polarized differentiation promoted the maturity and liver function of human embryonic stem cell-derived hepatocytes via activating Hippo and AMPK signaling pathways. Cells. 2022;11(24): Article 4117. 36552880 10.3390/cells11244117 PMC9776724

[11] Hughes CS, Postovit LM, Lajoie GA. Matrigel: A complex protein mixture required for optimal growth of cell culture. Proteomics. 2010;10(9):1886–1890. 20162561 10.1002/pmic.200900758

[12] Hendriks D, Brouwers JF, Hamer K, Geurts MH, Luciana L, Massalini S, López-Iglesias C, Peters PJ, Rodríguez-Colman MJ, Chuva de Sousa Lopes S, et al. Engineered human hepatocyte organoids enable CRISPR-based target discovery and drug screening for steatosis. Nat Biotechnol. 2023;41(11):1567–1581. 36823355 10.1038/s41587-023-01680-4 PMC10635827

[13] Hendriks D, Artegiani B, Hu H, Chuva de Sousa Lopes S, Clevers H. Establishment of human fetal hepatocyte organoids and CRISPR–Cas9-based gene knockin and knockout in organoid cultures from human liver. Nat Protoc. 2021;16(1):182–217. 33247284 10.1038/s41596-020-00411-2

[14] Sorrentino G, Rezakhani S, Yildiz E, Nuciforo S, Heim MH, Lutolf MP, Schoonjans K. Mechano-modulatory synthetic niches for liver organoid derivation. Nat Commun. 2020;11(1):3416. 32651372 10.1038/s41467-020-17161-0 PMC7351772

[15] Ye S, Boeter JW, Mihajlovic M, van Steenbeek FG, van Wolferen ME, Oosterhoff LA, Marsee A, Caiazzo M, van der Laan LJ, Penning LC, et al. A chemically defined hydrogel for human liver organoid culture. Adv Funct Mater. 2020;30(48):2000893. 34658689 10.1002/adfm.202000893 PMC7611838

[16] Gjorevski N, Sachs N, Manfrin A, Giger S, Bragina ME, Ordóñez-Morán P, Clevers H, Lutolf MP. Designer matrices for intestinal stem cell and organoid culture. Nature. 2016;539(7630):560–564. 27851739 10.1038/nature20168

[17] Liu H, Wang Y, Cui K, Guo Y, Zhang X, Qin J. Advances in hydrogels in organoids and organs-on-a-chip. Adv Mater. 2019;31(50): Article e1902042. 31282047 10.1002/adma.201902042

[18] Wu H, Wang J, Liu S, Wang Y, Tang X, Xie J, Wang N, Shan H, Chen S, Zhang X, et al. Large-scale production of expandable hepatoblast organoids and polarised hepatocyte organoids from hESCs under 3D static and dynamic suspension conditions. Cell Prolif. 2025;58(7): Article e70001. 39921573 10.1111/cpr.70001 PMC12240639

[19] Sahabian A, Dahlmann J, Martin U, Olmer R. Production and cryopreservation of definitive endoderm from human pluripotent stem cells under defined and scalable culture conditions. Nat Protoc. 2021;16(3):1581–1599. 33580232 10.1038/s41596-020-00470-5

[20] Tang X, Wu H, Xie J, Wang N, Chen Q, Zhong Z, Qiu Y, Wang J, Li X, Situ P, et al. The combination of dextran sulphate and polyvinyl alcohol prevents excess aggregation and promotes proliferation of pluripotent stem cells in suspension culture. Cell Prolif. 2021;54(9): Article e13112. 34390064 10.1111/cpr.13112 PMC8450127

[21] Pan T, Tao J, Chen Y, Zhang J, Getachew A, Zhuang Y, Wang N, Xu Y, Tan S, Fang J, et al. Robust expansion and functional maturation of human hepatoblasts by chemical strategy. Stem Cell Res Ther. 2021;12(1):151. 33632328 10.1186/s13287-021-02233-9 PMC7908723

[22] Pan T, Chen Y, Zhuang Y, Yang F, Xu Y, Tao J, You K, Wang N, Wu Y, Lin X, et al. Synergistic modulation of signaling pathways to expand and maintain the bipotency of human hepatoblasts. Stem Cell Res Ther. 2019;10(1):364. 31791391 10.1186/s13287-019-1463-y PMC6888929

[23] Lee J, Cuddihy MJ, Kotov NA. Three-dimensional cell culture matrices: State of the art. Tissue Eng Part B Rev. 2008;14(1):61–86. 18454635 10.1089/teb.2007.0150

[24] Wang Y, Kim MH, Shirahama H, Lee JH, Ng SS, Glenn JS, Cho NJ. ECM proteins in a microporous scaffold influence hepatocyte morphology, function, and gene expression. Sci Rep. 2016;6(1):37427. 27897167 10.1038/srep37427 PMC5126637

[25] Liu S, Yao S, Yang H, Liu S, Wang Y. Autophagy: Regulator of cell death. Cell Death Dis. 2023;14(10):648. 37794028 10.1038/s41419-023-06154-8 PMC10551038

[26] Glick D, Barth S, Macleod KF. Autophagy: Cellular and molecular mechanisms. J Pathol. 2010;221(1):3–12. 20225336 10.1002/path.2697 PMC2990190

[27] Filomeni G, De Zio D, Cecconi F. Oxidative stress and autophagy: The clash between damage and metabolic needs. Cell Death Differ. 2015;22(3):377–388. 25257172 10.1038/cdd.2014.150 PMC4326572

[28] Gonzalez-Meljem JM, Apps JR, Fraser HC, Martinez-Barbera JP. Paracrine roles of cellular senescence in promoting tumourigenesis. Br J Cancer. 2018;118(10):1283–1288. 29670296 10.1038/s41416-018-0066-1 PMC5959857

[29] Kim YC, Guan KL. mTOR: A pharmacologic target for autophagy regulation. J Clin Invest. 2015;125(1):25–32. 25654547 10.1172/JCI73939 PMC4382265

[30] Treyer A, Müsch A. Hepatocyte polarity. Compr Physiol. 2013;3(1):243–287. 23720287 10.1002/cphy.c120009 PMC3697931

[31] Dao Thi VL, Wu X, Belote RL, Andreo U, Takacs CN, Fernandez JP, Vale-Silva LA, Prallet S, Decker CC, Fu RM, et al. Stem cell-derived polarized hepatocytes. Nat Commun. 2020;11(1):1677. 32245952 10.1038/s41467-020-15337-2 PMC7125181

[32] Co JY, Margalef-Català M, Monack DM, Amieva MR. Controlling the polarity of human gastrointestinal organoids to investigate epithelial biology and infectious diseases. Nat Protoc. 2021;16(11):5171–5192. 34663962 10.1038/s41596-021-00607-0 PMC8841224

[33] Serras AS, Rodrigues JS, Cipriano M, Rodrigues AV, Oliveira NG, Miranda JP. A critical perspective on 3D liver models for drug metabolism and toxicology studies. Front Cell Dev Biol. 2021;9: Article 626805. 33732695 10.3389/fcell.2021.626805 PMC7957963

[34] Gómez-Lechón MJ, Tolosa L, Donato MT. Upgrading HepG2 cells with adenoviral vectors that encode drug-metabolizing enzymes: Application for drug hepatotoxicity testing. Expert Opin Drug Metab Toxicol. 2017;13(2):137–148. 27671376 10.1080/17425255.2017.1238459

[35] Jung DJ, Byeon JH, Jeong GS. Flow enhances phenotypic and maturation of adult rat liver organoids. Biofabrication. 2020;12(4): Article 045035. 33000764 10.1088/1758-5090/abb538

[36] Li F, Cao L, Parikh S, Zuo R. Three-dimensional spheroids with primary human liver cells and differential roles of Kupffer cells in drug-induced liver injury. J Pharm Sci. 2020;109(6):1912–1923. 32145211 10.1016/j.xphs.2020.02.021

[37] Zamek-Gliszczynski MJ, Xiong H, Patel NJ, Turncliff RZ, Pollack GM, Brouwer KL. Pharmacokinetics of 5 (and 6)-carboxy-2′,7′-dichlorofluorescein and its diacetate promoiety in the liver. J Pharmacol Exp Ther. 2003;304(2):801–809. 12538836 10.1124/jpet.102.044107

[38] Shinozawa T, Kimura M, Cai Y, Saiki N, Yoneyama Y, Ouchi R, Koike H, Maezawa M, Zhang RR, Dunn A, et al. High-fidelity drug-induced liver injury screen using human pluripotent stem cell–derived organoids. Gastroenterology. 2021;160(3):831–846.e10. 33039464 10.1053/j.gastro.2020.10.002 PMC7878295

[39] Fu D, Wakabayashi Y, Lippincott-Schwartz J, Arias IM. Bile acid stimulates hepatocyte polarization through a cAMP-Epac-MEK-LKB1-AMPK pathway. Proc Natl Acad Sci USA. 2011;108(4):1403–1408. 21220320 10.1073/pnas.1018376108 PMC3029747

[40] Yu W, Datta A, Leroy P, O’Brien LE, Mak G, Jou TS, Matlin KS, Mostov KE, Zegers MM. β1-Integrin orients epithelial polarity via Rac1 and laminin. Mol Biol Cell. 2005;16(2):433–445. 15574881 10.1091/mbc.E04-05-0435 PMC545874

[41] Makishima M. Nuclear receptors as targets for drug development: Regulation of cholesterol and bile acid metabolism by nuclear receptors. J Pharmacol Sci. 2005;97(2):177–183. 15725701 10.1254/jphs.fmj04008x4

[42] Wu H, Tang X, Wang Y, Wang N, Chen Q, Xie J, Liu S, Zhong Z, Qiu Y, Situ P, et al. Dextran sulfate prevents excess aggregation of human pluripotent stem cells in 3D culture by inhibiting ICAM1 expression coupled with down-regulating E-cadherin through activating the Wnt signaling pathway. Stem Cell Res Ther. 2022;13(1):218. 35619172 10.1186/s13287-022-02890-4 PMC9137216

[43] Kriedemann N, Triebert W, Teske J, Mertens M, Franke A, Ullmann K, Manstein F, Drakhlis L, Haase A, Halloin C, et al. Standardized production of hPSC-derived cardiomyocyte aggregates in stirred spinner flasks. Nat Protoc. 2024;19(7):1911–1939. 38548938 10.1038/s41596-024-00976-2

[44] Iworima DG, Baker RK, Piret JM, Kieffer TJ. Analysis of the effects of bench-scale cell culture platforms and inoculum cell concentrations on PSC aggregate formation and culture. Front Bioeng Biotechnol. 2023;11:1267007. 38107616 10.3389/fbioe.2023.1267007 PMC10722899

[45] Li X, Ma R, Gu Q, Liang L, Wang L, Zhang Y, Wang X, Liu X, Li Z, Fang J, et al. A fully defined static suspension culture system for large-scale human embryonic stem cell production. Cell Death Dis. 2018;9(9):892. 30166524 10.1038/s41419-018-0863-8 PMC6117302

[46] Giobbe GG, Crowley C, Luni C, Campinoti S, Khedr M, Kretzschmar K, De Santis MM, Zambaiti E, Michielin F, Meran L, et al. Extracellular matrix hydrogel derived from decellularized tissues enables endodermal organoid culture. Nat Commun. 2019;10(1):5658. 31827102 10.1038/s41467-019-13605-4 PMC6906306

[47] Willemse J et al. Hydrogels derived from decellularized liver tissue support the growth and differentiation of cholangiocyte organoids. Biomaterials. 2022;284: Article 121473. 35344800 10.1016/j.biomaterials.2022.121473

[48] Willemse J, van Tienderen G, van Hengel E, Schurink I, van der Ven D, Kan Y, de Ruiter P, Rosmark O, Schneeberger K, van der Eerden B, Roest H. β1 integrin/FAK/ERK signalling pathway is essential for human fetal islet cell differentiation and survival. J Pathol. 2009;219(2):182–192. 19544355 10.1002/path.2577

[49] Jiang X, Huang X, Zheng G, Jia G, Li Z, Ding X, Lei L, Yuan L, Xu S, Gao N. Targeting PI4KA sensitizes refractory leukemia to chemotherapy by modulating the ERK/AMPK/OXPHOS axis. Theranostics. 2022;12(16):6972–6988. 36276647 10.7150/thno.76563 PMC9576605

[50] Yu Z, Wu K, Fan C, Wang J, Chu F, He W, Ji Z, Deng Y, Hua D, Zhang Y, et al. Viscoelastic hydrogel promotes disc mechanical homeostasis repair and delays intervertebral disc degeneration via the yes-associated protein pathway. Biomater Res. 2025;29:0150. 40040957 10.34133/bmr.0150 PMC11876543

