# Supplementary material for: Distinct Roles of Matrigel Enabled the Production of Expandable Hepatoblast and Polarized Hepatocyte Organoids from Human Embryonic Stem Cells under 3-Dimensional Suspension Conditions
Source: Biomater Res. 2025 Nov 7;29:0280. doi: 10.34133/bmr.0280 (PMC12592637; doi:10.34133/bmr.0280)
Supplement: Supplementary 1 — Figs. S1 to S4 Tables S1 to S4 [file bmr.0280.f1.docx]

Supporting Information

**Distinct roles of Matrigel enabled productions of expandable hepatoblast and polarized hepatocyte organoids from hESCs under 3D suspension conditions**

Haibin Wu^1,2,3†^, Jue Wang^3†^, Shoupei Liu^1,2,3^, Yiyu Wang^2,3^, Jinghe Xie^3,4^, Xueyan Zhang^2,3^, Shuai Zhang^5^, Weili Gu^5,6^, Yongjian Zhou^1^, Yuyou Duan^1,2,3,7,8^*

**Affiliations:**

^1^Department of Gastroenterology and Hepatology, Guangzhou Digestive Disease Center, the Second Affiliated Hospital, School of Medicine, South China University of Technology; Guangzhou, 510006, China.

^2^Laboratory of Stem Cells and Translational Medicine, Center for medical research on innovation and translation, Institute of Clinical Medicine, the Second Affiliated Hospital, School of Medicine, South China University of Technology; Guangzhou, 510006, China.

^3^Laboratory of Stem Cells and Translational Medicine, Institute for Life Science, School of Medicine, South China University of Technology; Guangzhou, 510006, China.

^4^School of Biomedical Sciences and Engineering, South China University of Technology, Guangzhou International Campus; Guangzhou, 511442, China.

^5^Department of Gastroenterology and Hepatology, Guangzhou Digestive Disease Center, Guangzhou First People’s Hospital, Guangzhou 510180, China.

^6^Surgical Department of Hepatobiliary Pancreas and Spleen, Affiliated Chinese Medicine Hospital at Tianhe Campus, Guangzhou Medical University, Guangzhou 510645, P.R. China.

^7^National Engineering Research Center for Tissue Restoration and Reconstruction, South China University of Technology; Guangzhou, 510006, China.

^8^The Innovation Centre of Ministry of Education for Development and Diseases, the Second Affiliated Hospital, School of Medicine, South China University of Technology; Guangzhou 510006, China.

* **Correspondence to:**

Yuyou Duan, Ph.D.

Laboratory of Stem Cells and Translational Medicine

Institute for Clinical Medicine

Second Affiliation Hospital

School of Medicine

South China University of Technology

No. 10 Huanyu Erlu

Guangzhou 510005, China

E-mail: [yuyouduan@scut.edu.cn](mailto:yuyouduan@scut.edu.cn)

†These authors contributed equally to this work.

**Supplementary Figures**


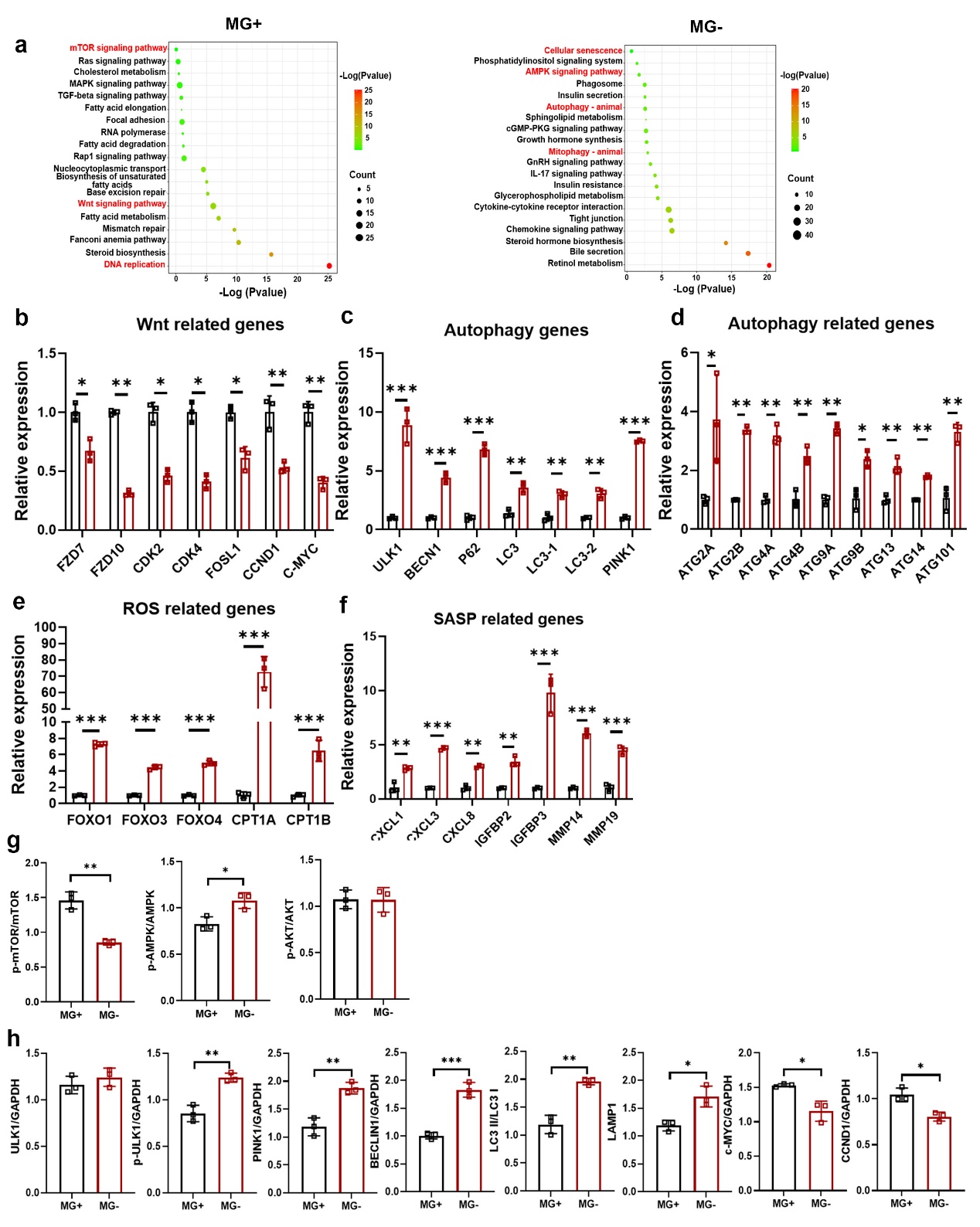


**Figure S1. Characterizations of HB-orgs cultured with Matrigel (MG+) or without Matrigel (MG-)**

(a) KEGG analysis of HB-orgs cultured with Matrigel (MG+) or without Matrigel (MG-). (b-f) The relative expression levels of Wnt pathway-related genes (n = 3 independent experiments) (b), autophagy genes (n = 3 independent experiments) (c), autophagy-related genes (n = 3 independent experiments) (d), ROS-related genes (n = 3 independent experiments) (e) and SASP-related genes (n = 3 independent experiments) (f) were determined by qRT-PCR in HB-orgs cultured with or without Matrigel (n = 3 for each group). (g, h) Densitometric analysis of WB band intensities in Fig. 2H and 2I from three independent experiments (n = 3). Results were presented as mean ± SD. **p* <0.05, ***p* <0.01 and ****p* <0.001.


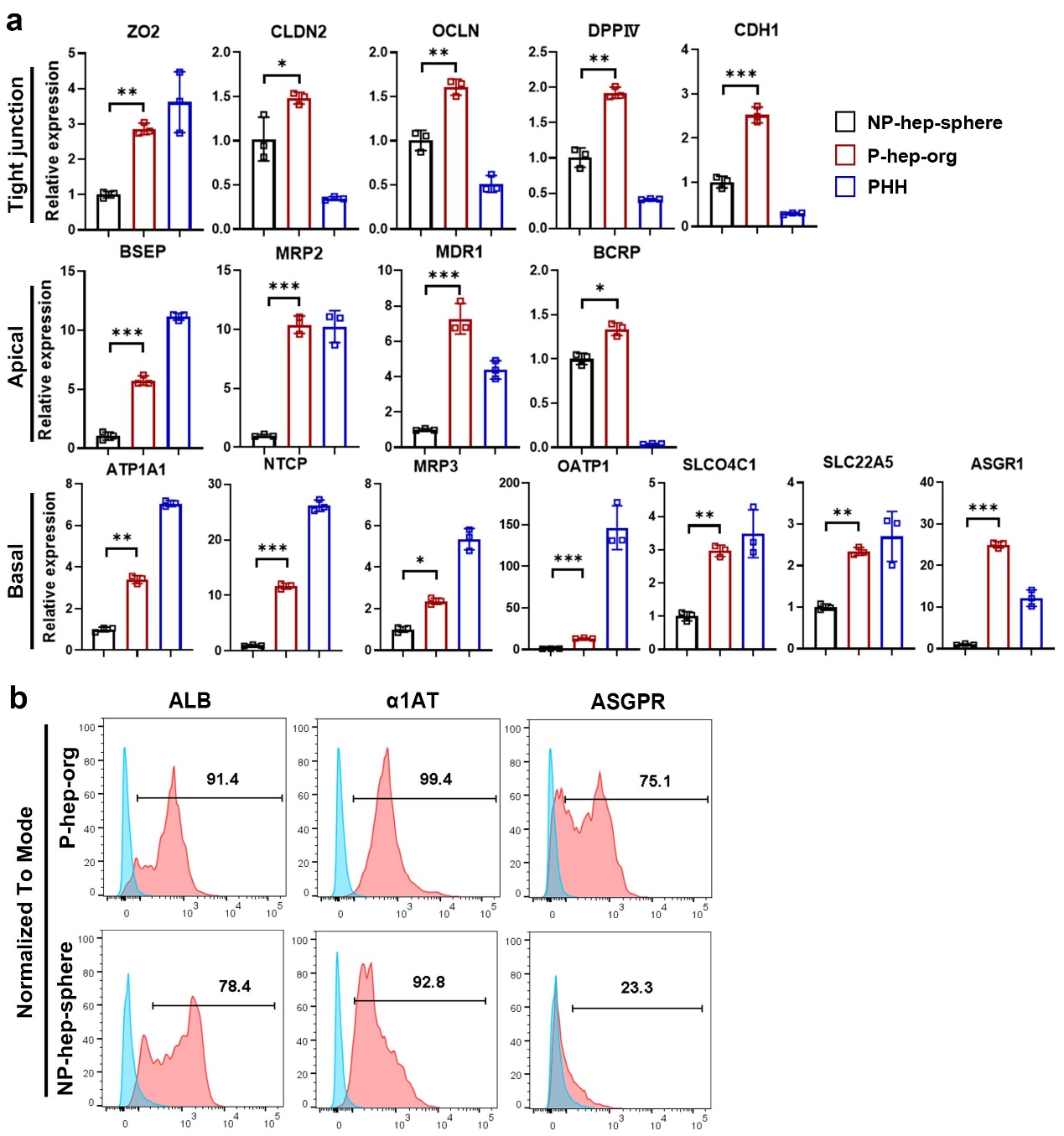


**Figure S2. Characterizations of P-hep-orgs and NP-hep-spheres**

(a) The relative expression levels of a large number of polarity-related genes including tight junction genes (top panel), apical genes (middle panel), and basal genes (bottom panel) were analyzed by qRT-PCR among NP-hep-spheres, P-hep-orgs and PHHs (n = 3 independent experiments). Results were presented as mean ± SD. (b) Percentages of positive cells for mature hepatocyte markers were examined in P-hep-orgs or NP-hep-spheres by flow cytometry. **p* <0.05, ***p* <0.01 and ****p* <0.001.


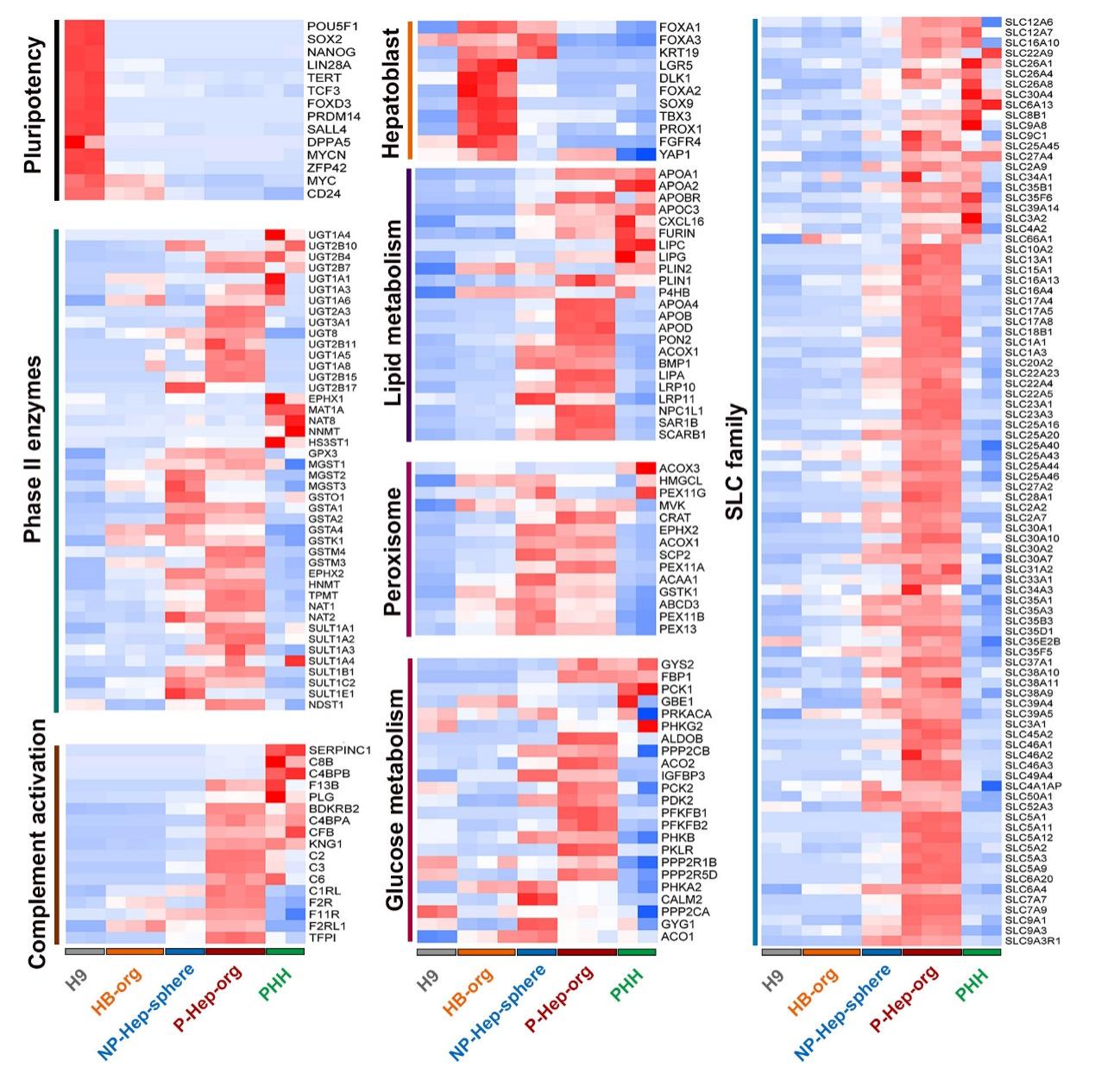


**Figure S3. Heatmap of P-hep-orgs, NP-hep-spheres and PHHs for genes related to the pluripotency, phase II enzymes, complement activation, hepatoblast, lipid, glucose metabolism, peroxisome and SLC family**


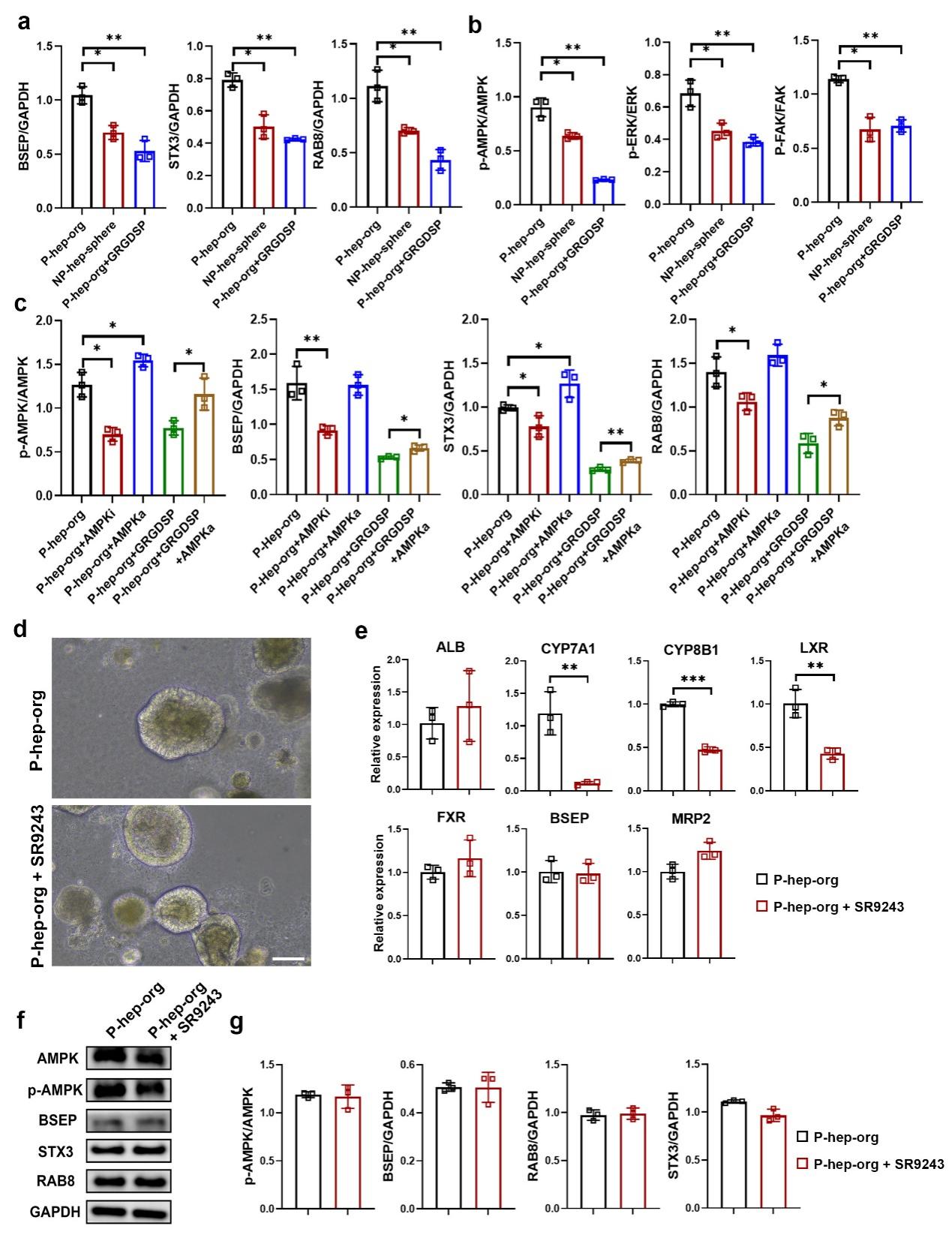


**Figure S4. Investigation on the relationship between the polarization of P-hep-orgs and bile acid**

(a-c) Densitometric analysis of WB band intensities in Fig. 7D-F from three independent experiments (n = 3). (d) Representative morphologies of P-hep-orgs treated with or without bile acid inhibitor (1 μM SR9242), scale bar = 100 μm. (e) The relative expression levels of genes associated with bile acid synthesis (CYP7A1, CYP8B1), modulation (LXR, FXR) and secretion (BSEP, MRP2) were analyzed by qRT-PCR among different cells (n = 3 independent experiments). (f) Western blotting analysis for the expression of polarization-related proteins AMPK, p-AMPK, BSEP, STX3 and RAB8 after the treatment with or without SR9242 in P-hep-orgs. (g) Densitometric analysis of WB band intensities in Fig. S4f from three independent experiments (n = 3). Results were presented as mean ± SD. ***p* <0.01 and ****p* <0.001.

**Table S1. Primers used in this study**

| Gene | Forward | Reverse |
| --- | --- | --- |
| GAPDH | GAAGATGGTGATGGGATTTC | GAAGGTGAAGGTCGGAGTC |
| ALB | CTGCCTGCCTGTTGCCAAAGC | GGCAAGGTCCGCCCTGTCATC |
| FZD7 | GTCTTCAGCGTGCTCTACACAG | ACGGCATAGCTCTTGCACGTCT |
| FZD10 | GAACACGGACAAGCTGGAGAAG | GGCGTTCGTAAAAGTAGCAGGC |
| CDK2 | ATGGATGCCTCTGCTCTCACTG | CCCGATGAGAATGGCAGAAAGC |
| CDK4 | CCATCAGCACAGTTCGTGAGGT | TCAGTTCGGGATGTGGCACAGA |
| FOSL1 | GGAGGAAGGAACTGACCGACTT | CTCTAGGCGCTCCTTCTGCTTC |
| CCND1 | TCTACACCGACAACTCCATCCG | TCTGGCATTTTGGAGAGGAAGTG |
| c-MYC | AGCTCATTTCTGAAGAGGACTTGT | TTGAGGCAGTTTACATTATGGCTA |
| ULK1 | GCAAGGACTCTTCCTGTGACAC | CCACTGCACATCAGGCTGTCTG |
| BECN1 | CTGGACACTCAGCTCAACGTCA | CTCTAGTGCCAGCTCCTTTAGC |
| P62 | TGTGTAGCGTCTGCGAGGGAAA | AGTGTCCGTGTTTCACCTTCCG |
| LC3 | ATCTCCGAGCTGAGGATGCCTT | GACACTTTCGTCACTGTAGGCAA |
| LC3-1 | TTGTAGAGAAGGCTCCAAAAGCC | GGTCTCAGGTGGATTCTCTTCC |
| LC3-2 | CCAGCTTCCTTCTGAAAAGGCG | TTCTCTCCGCTGTAGGCCACAT |
| PINK1 | GTGGACCATCTGGTTCAACAGG | GCAGCCAAAATCTGCGATCACC |
| ATG2A | GGTAGAACTCCAAGGACATCTGC | CCAGACAGAAGTAGCCAAGTCC |
| ATG2B | CTTCAGATGGAGTTGGAGGAGAC | AGTGGCTCCTTTCAGTCCTACG |
| ATG4A | CCAAGCCAGAAGTGACAACCAC | GACAGACCTTCAAGTTGAGTTCC |
| ATG4B | ATGGGAGTTGGCGAAGGCAAGT | AGCTCCACGTATCGAAGACAGC |
| ATG9A | GCTTCCTCAAGGAGCAGGTTCA | CCACATTTGCGATAAGGCTCAGG |
| ATG9B | ACCCTGTCAGATGCCATCCTAC | CCAGTAGCTGAAGAGGTTGCAG |
| ATG13 | CAGAACTGCTGGTGAGGACACT | AGCAGGCTGATAGGAAAGGCGA |
| ATG14 | TGTACCTGGTCAGTCCAAGCTC | CAGGTCGGTTTCTTCATCGCTG |
| ATG101 | GCGTGTCTCTTCTGAGGAACTG | GGTAGAACTCCAAGGACATCTGCC |
| FOXO1 | CTACGAGTGGATGGTCAAGAGC | CCAGTTCCTTCATTCTGCACACG |
| FOXO3 | TCTACGAGTGGATGGTGCGTTG | CTCTTGCCAGTTCCCTCATTCTG |
| FOXO4 | ACGAGTGGATGGTCCGTACTGT | CCTTGATGAACTTGCTGTGCAGGC |
| CPT1A | GATCCTGGACAATACCTCGGAGC | CTCCACAGCATCAAGAGACTGC |
| CPT1B | TGTATCGCCGTAAACTGGACCG | TGTCTGAGAGGTGCTGTAGCAC |
| CXCL1 | AGCTTGCCTCAATCCTGCATCC | TCCTTCAGGAACAGCCACCAGT |
| CXCL3 | TTCACCTCAAGAACATCCAAAGTG | TTCTTCCCATTCTTGAGTGTGGC |
| CXCL8 | GAGAGTGATTGAGAGTGGACCAC | CACAACCCTCTGCACCCAGTTT |
| IGFBP2 | CGAGGGCACTTGTGAGAAGCG | TGTTCATGGTGCTGTCCACGTG |
| IGFBP3 | CGCTACAAAGTTGACTACGAGTC | GTCTTCCATTTCTCTACGGCAGG |
| MMP14 | CCTTGGACTGTCAGGAATGAGG | TTCTCCGTGTCCATCCACTGGT |
| MMP19 | GCAGTAGTGAACTGGATGCCATG | CAAAGGGCAGACACTCGGAACA |
| FXR | GGTCTCGTAGACGAAGGACTGA | TGTCTGCTCTGAGACTCAGCTC |
| CPS1 | CTAGCCTGGATTACATGGTCACC | CCTCAAAGGTACGACCAATAGCC |
| ASL | CTCTCAACAGCATGGATGCCAC | CTTGGTGCAGTAGAGGATGAGG |
| NAGS | CAGTTCCAGACCTGCCATCACT | ATGTCCATGCGCTGCAAGAAGG |
| ASS1 | GCTGAAGGAACAAGGCTATGACG | GCCAGATGAACTCCTCCACAAAC |
| OTC | ATCCTGGCTGATTACCTCACGC | CCGAATTTCGCTGCGCTCATCA |
| LXR | TGGACACCTACATGCGTCGCAA | CAAGGATGTGGCATGAGCCTGT |
| CYP7A1 | CAAGCAAACACCATTCCAGCGAC | ATAGGATTGCCTTCCAAGCTGAC |
| CYP8B1 | CTGGAGACCAAGCAGTCCTTTG | GATACTCCTGCCCACTGGACAT |
| ZO2 | ATTAGTGCGGGAGGATGCCGTT | TCTGCCACAAGCCAGGATGTCT |
| CLDN2 | ATGGCAAAGTGAATGACAAGCG | GGAGATTGCACTGGATGTCACC |
| OCLN | ATGGCAAAGTGAATGACAAGCGG | CTGTAACGAGGCTGCCTGAAGT |
| DPPIV | AAAGGCACCTGGGAAGTCATCG | CAGCTCACAACTGAGGCATGTC |
| CDH1 | CCCACCACGTACAAGGGTC | CTGGGGTATTGGGGGCATC |
| BSEP | AGCCACACAGACCAGGATGTTG | CAATGAACCGCCTCTCCTTTCC |
| MRP2 | GCCAACTTGTGGCTGTGATAGG | ATCCAGGACTGCTGTGGGACAT |
| MDR1 | TTGCTGCTTACATTCAGGTTTCA | AGCCTATCTCCTGTCGCATTA |
| BCRP | GTTCTCAGCAGCTCTTCGGCTT | TCCTCCAGACACACCACGGATA |
| ATP1A1 | GGCAGTGTTTCAGGCTAACCAG | TCTCCTTCACGGAACCACAGCA |
| NTCP | GCTCTCTTCTGCCTCAATGGAC | AGTGGTCCAATGACTTCAGGTGG |
| MRP3 | GAGGAGAAAGCAGCCATTGGCA | TCCAATGGCAGCCGCACTTTGA |
| OATP1 | GCGGAAATGCACCAGTTGAAGG | GGTTCTTCAGCAGGAGCCAGAT |
| SLCO4C1 | TGAGCCATTTGCTGGTGTATCTG | TGGACTCCATCTCCACAGACAG |
| SLC22A5 | GCTACATGGTGCTGCCACTGTT | CTGCCTCTTCAAATCGTCCCTG |
| ASGR1 | GAAGCAGTTCGTGTCTGACCTG | AGCGAGAGAACCAGTAGCAGCT |

**Table S2. Antibodies used in this study**

| Primary antibody | | | | |
| --- | --- | --- | --- | --- |
| Antibody | Company | Product code | Ig Species | Dilution |
| Anti-GAPDH | Abcam | ab128915 | Rabbit | 10000 |
| Anti-HNF4a | CST | 3113 | Rabbit | 2000 |
| Anti-ALB | Bethyl | A80-129A | Goat | 1000 |
| Anti-AFP | Thermo | MIA1305 | Mouse | 200 |
| Anti-ZO1 | CST | 13663 | Rabbit | 200 |
| Anti-MDR1 | CST | 13342 | Rabbit | 200 |
| Anti-SLC10A1(NTCP) | Boster | PB9745 | Rabbit | 400 |
| Anti-mTOR | CST | 2983 | Rabbit | 1000 |
| Anti-p-mTOR | CST | 5536 | Rabbit | 1000 |
| Anti-AMPK | Proteintech | 10929-2-AP | Rabbit | 1000 |
| Anti-p-AMPK | CST | 2535 | Rabbit | 1000 |
| Anti-AKT | CST | 4691 | Rabbit | 1000 |
| Anti-p-AKT | CST | 4060 | Rabbit | 1000 |
| Anti-ULK1 | CST | 8054 | Rabbit | 1000 |
| Anti-p-ULK1 | CST | 5869 | Rabbit | 1000 |
| Anti-PINK1 | CST | 6946 | Rabbit | 1000 |
| Anti-BECLIN1 | CST | 3495 | Rabbit | 1000 |
| Anti-LC3A/B | CST | 12741 | Rabbit | 1000 |
| Anti-LAMP1 | CST | 9091 | Rabbit | 1000 |
| Anti-c-Myc | Bioss | bs-24507R | Rabbit | 1000 |
| Anti-CCND1 | CST | 55506 | Rabbit | 1000 |
| Anti-FAK | CST | 71433 | Rabbit | 1000 |
| Anti-p-FAK | CST | 8556 | Rabbit | 1000 |
| Anti-ERK | CST | 4695 | Rabbit | 1000 |
| Anti-p-ERK | CST | 4370 | Rabbit | 1000 |
| Anti-BSEP | Boster | PB9414 | Rabbit | 500 |
| Anti-STX3 | Abclonal | A3712 | Rabbit | 500 |
| Anti-RAB8 | CST | 6975 | Rabbit | 1000 |
| Human Serum Albumin PE-conjugated Antibody | R&D | IC1455P |  | 10 μL for million cells |
| PE anti-human ASGPR1 antibody | BD | 563655 |  | 5 μL for million cells |
| Goat anti-Human-A1AT（FITC） antibody | Bethyl | A80-122F |  | 1000 |
| Anti-Factin (Phalloidin-iFluor 594) | Abcam | ab176757 |  |  |
| Secondary antibody | | | |  |
| Secondary antibody | Company | Code number | Dilution |  |
| Alexa Fluor 488-conjugated goat anti-Rabbit IgG | CST | 4412 | 800 |  |
| Alexa Fluor 594-conjugated goat anti-Mouse IgG | CST | 8890 | 800 |  |
| Alexa Fluor 594-conjugated donkey anti-Goat IgG | Abcam | ab150136 | 800 |  |
| anti-rabbit IgG, HRP-linked antibody | CST | 7074 | 3000 |  |

**Table S3. TC_50_ of compounds in P-hep-org, NP-hep-sphere, and HepG2-3D**

| Toxin | P-hep-org (μM) | NP-hep-sphere (μM) | HepG2-3D (μM) |
| --- | --- | --- | --- |
| Mannitol | N.D | N.D | N.D |
| Troglitazone | 25.2±1.7 | 53.6±23.6 | 199.4±12.4 |
| Chlorpromazine | 0.1±0.02 | 1.2±0.2 | 84.1±9.3 |
| Dicofenac | 62.1±5.9 | 79.9±34.8 | 615.8±2.0 |
| Cyclosporine A | 11.9±2.4 | 16.1±2.6 | 2005.0±4.9 |
| Nefazodone | 16.4±3.2 | 36.3±1.2 | 63.1±6.0 |
| Tolcapone | 48.6±2.1 | 50.3±7.5 | 720.9±13.4 |
| Bosentan | 81.8±6.2 | 148.2±33.5 | 643.6±2.2 |

**Table S4. Comparative MOS (TC_50_/C_max_) values across P-hep-org, NP-hep-sphere, and HepG2-3D models**

| Toxin | P-hep-org | NP-hep-sphere | HepG2-3D |
| --- | --- | --- | --- |
| Troglitazone | 7.00 | 14.89 | 55.39 |
| Chlorpromazine | 0.11 | 1.33 | 93.44 |
| Dicofenac | 7.76 | 9.99 | 76.98 |
| Cyclosporine A | 19.83 | 26.83 | 3341.67 |
| Nefazodone | 3.57 | 7.89 | 13.72 |
| Tolcapone | 2.21 | 2.29 | 32.77 |
| Bosentan | 11.05 | 20.03 | 86.97 |

Human Cmax values: Troglitazone = 3.6 μM[1], Chlorpromazine = 0.9 μM[2], Dicofenac = 8 μM[3], Cyclosporine A = 0.6 μM[4], Nefazodone = 4.3 μM[2], Tolcapone = 22 μM[5], Bosentan = 7.4 μM[6]. The MOS values were calculated based on the average TC₅₀ values.

1. Loi, C.M., et al., *Steady-state pharmacokinetics and dose proportionality of troglitazone and its metabolites.* J Clin Pharmacol, 1999. **39**(9): p. 920-6.

2. Li, F., et al., *Three-Dimensional Spheroids With Primary Human Liver Cells and Differential Roles of Kupffer Cells in Drug-Induced Liver Injury.* J Pharm Sci, 2020. **109**(6): p. 1912-1923.

3. G.D. Searle LLC, D.o.P.I. *FDA professional drug information – Arthrotec*. 2015 [cited 2016 April 3]; Available from: <http://www.drugs.com/pro/arthrotec.html>.

4. Fu, L.W., et al., *Cyclosporin pharmacokinetics following administration of capsules and Neoral in paediatric patients with lupus nephritis.* Br J Clin Pharmacol, 1997. **44**(2): p. 125-7.

5. Jorga, K., et al., *Metabolism and excretion of tolcapone, a novel inhibitor of catechol-O-methyltransferase.* Br J Clin Pharmacol, 1999. **48**(4): p. 513-20.

6. Gutierrez, M.M., et al., *Relative bioavailability of a newly developed pediatric formulation of bosentan vs. the adult formulation.* Int J Clin Pharmacol Ther, 2013. **51**(6): p. 529-36.
